# Drop-cast gold nanoparticles are not always electrocatalytically active for the borohydride oxidation reaction†

**DOI:** 10.1039/d4sc00676c

**Published:** 2024-04-11

**Authors:** Lachlan F. Gaudin, Alison M. Funston, Cameron L. Bentley

**Affiliations:** a School of Chemistry, Monash University Clayton 3800 VIC Australia cameron.bentley@monash.edu; b ARC Centre of Excellence in Exciton Science, Monash University Clayton 3800 VIC Australia

## Abstract

The next-generation of energy devices rely on advanced catalytic materials, especially electrocatalytic nanoparticles (NPs), to achieve the performance and cost required to reshape the energy landscape towards a more sustainable and cleaner future. It has become imperative to maximize the performance of the catalyst, both through improvement of the intrinsic activity of the NP, and by ensuring all particles are performing at the level of their capability. This requires not just a structure–function understanding of the catalytic material, but also an understanding of how the catalyst performance is impacted by its environment (substrate, ligand, *etc.*). The intrinsic activity and environment of catalytic particles on a support may differ wildly by particle, thus it is essential to build this understanding from a single-entity perspective. To achieve this herein, scanning electrochemical cell microscopy (SECCM) has been used, which is a droplet-based scanning probe technique which can encapsulate single NPs, and apply a voltage to the nanoparticle whilst measuring its resulting current. Using SECCM, single AuNPs have been encapsulated, and their activity for the borohydride oxidation reaction (BOR) is measured. A total of 268 BOR-active locations were probed (178 single particles) and a series of statistical analyses were performed in order to make the following discoveries: (1) a certain percentage of AuNPs display no BOR activity in the SECCM experiment (67.4% of single NPs), (2) visibly-similar particles display wildly varied BOR activities which cannot be explained by particle size, (3) the impact of cluster size (#NP at a single location) on a selection of diagnostic electrochemical parameters can be easily probed with SECCM, (4) exploratory statistical correlation between these parameters can be meaningfully performed with SECCM, and (5) outlying “abnormal” NP responses can be probed on a particle-by-particle basis. Each one of these findings is its own worthwhile study, yet this has been achieved with a single SECCM scan. It is hoped that this research will spur electrochemists and materials scientists to delve deeper into their substantial datasets in order to enhance the structure–function understanding, to bring about the next generation of high-performance electrocatalysts.

## Abstract 2

A collection of single Au nanoparticles (NPs) and NP clusters were individually tested for the borohydride oxidation reaction. Significant variation in the activity could not be attributed to NP size, and ∼67% of NP locations displayed no activity.

## Graphical abstract

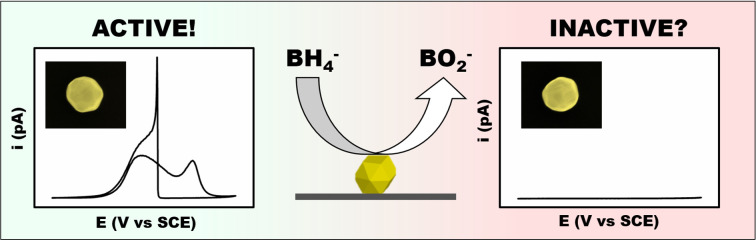

## 1 Introduction

Achieving a successful energy transition is a central goal of many avenues of research across the chemical and materials sciences.^1,2^ The development and refinement of new energy materials, especially those used in renewable fuel technologies (*e.g.* electrolysers and fuel cells), is pivotal to enable the transition away from the current reliance on unsustainable energy production/consumption methods.^3^ Many of these emerging technologies require electrochemical materials (*e.g.* electrocatalysts), and thus the advancement of these is a central focus for electrochemists.^4,5^ These new materials often face significant limitations, such as the stability of the electrode material,^6–8^ or prohibitive cost of the noble metals often required,^9,10^ which currently prevents their use in industrially-relevant applications. There is therefore an incentive for electrochemists to find alternatives to these materials, or to uncover ways of optimising their usage.

A model example of this is the development of fuel cells, wherein electricity is directly generated through the consumption of a chemical fuel.^11^ The efficiency of a fuel cell can be increased, and operating temperature decreased, with the use of a suitable electrocatalyst. A prominent example of a fuel cell which is currently being deployed in industry is the proton exchange membrane fuel cell (PEMFC).^12^ A developing category of next-generation fuel cells is based on the electro-oxidation of sodium borohydride (NaBH_4_) into sodium metaborate (NaBO_2_), in a process known as the borohydride oxidation reaction (BOR), which occurs readily in alkaline conditions with a suitable catalyst.^13^ This technology is termed the direct borohydride fuel cell (DBFC), and has higher operating voltage when compared with the PEMFC, providing the DBFC a niche when higher power density applications are required with fewer cells.^13^ Additionally, the expensive platinum catalysts which are the state-of-the-art for PEMFCs are not required for DBFCs.^13,14^ The BOR is also a highly suitable model process for the fundamental investigation of electrocatalytic materials due to the large number of electrons passed (8 e^−^ per complete reaction) for each direct electrochemical conversion of BH_4_^−^ to BO_2_^−^, generating high currents.^15^

An ongoing target for electrochemists is to minimise the cost of the often-expensive catalyst materials, which naturally leads to the deployment of nanomaterials.^16^ The use of nanomaterials with a high surface-area-to-volume-ratio can achieve similar geometric current densities with a fraction of the catalyst mass under operating conditions.^17^ Additionally, the intrinsic activity of nanomaterials is often higher than that of bulk materials, due to the higher surface energy of the nanomaterial.^18^ For these reasons, the current landscape of emerging electrocatalysts is dominated by advanced nanomaterials, with many of the most promising being based on nanoparticles (NPs).^19–21^

NP electrocatalysis has been traditionally challenging to study, as the only method of probing their catalytic performance has been by depositing them onto a surface and testing their activity in the aggregate.^22^ This method will summate the countless number of NPs present on the surface into an aggregated response, which will “smooth over” any interesting structure–function results that arise from the unique activities of individual NPs. Concerningly, with such an approach, there may be no way to tell the difference between a material with a narrow distribution of NPs possessing average activity, and a wide distribution of NP responses, some of which may be inactive or super-active catalysts. To overcome this, electrochemists have produced functionalised surfaces with highly-structured, monodisperse NPs.^23–25^ This has required careful synthesis and maintenance of the NPs,^26^ prevents investigation of local clustering effects,^27^ does not address the issue of potentially inactive/outlying particles,^28^ and often does not match the kind of material present in industrially-relevant devices.

In recent years, emphasis has been placed on investigating the catalytic activity of “single-entities”, with the goal of circumventing this issue.^29^ A benchmark tool for this new avenue of electrochemical research is the recently-developed scanning electrochemical cell microscopy (SECCM),^30^ where a nano/micropipette filled with electrolyte is approached to a surface, and a small droplet of the electrolyte is allowed to make contact, which is able to encapsulate and apply a potential to a single NP.^31–34^ This method of probing single-NP activity enables the investigation of single-NP catalysis, and post-experiment identical-location structural analyses using any number of techniques, including scanning electron microscopy (SEM),^35–38^ transmission electron microscopy (TEM),^39^ and atomic force microscopy (AFM).^40,41^ This enables the development of structure–function information in a high-throughput fashion with a single sample, and often within a single scan area, as demonstrated herein.

In this work, SECCM was deployed in the hopping-mode to encapsulate single Au NPs and small NP clusters (NPCs; comprised of 2–5 individual NPs) on a highly-ordered pyrolytic graphite (HOPG) substrate, and their cyclic voltammograms (CVs) were measured to determine their activity for the BOR. Single NPs and NPCs both demonstrated significant variation in BOR activity which could not be simply explained by NP size. The inclusion of NPCs of a range of sizes enabled the effect of cluster size to be determined for a range of electrochemical parameters. Furthermore, it was found that a significant portion of NP-containing locations on the surface showed no activity for the BOR. This study highlights the power of a single SECCM experiment to reveal multiple avenues of crucial electrochemical information about a NP-functionalised material for use in industrially-relevant applications.

## 2 Experimental

### 2.1 Preparation of electrolytes and electrodes

Sodium hydroxide (NaOH, ≥ 97%, Sigma-Aldrich, USA) and sodium borohydride (NaBH_4_, 99%, Sigma-Aldrich, USA) were used as supplied, and all solutions and washing steps mentioned herein used ultrapure deionised water (Direct-Q Water Purification System, Milli-Q, USA). A 5 mM solution of NaBH_4_ was made up in 0.1 M NaOH, and all electrochemistry herein was performed with this freshly prepared solution. A freshly-exfoliated surface of HOPG (Grade ZYA, SPI Supplies, USA) was prepared using the scotch-tape method. A suspension of AuNPs (∼200 nm diameter, 1.70 × 10^9^–2.10 × 10^9^ particles per mL, stabilised in a citrate buffer, Sigma-Aldrich) was first sonicated for 5 minutes, and then diluted with methanol (MeOH, UNIVAR, USA) to a 1 : 20 ratio (MeOH : AuNP stock). A drop of this suspension (20 μL) was deposited on the HOPG surface and left covered for 30 min. The droplet was washed off with water before being left in contact with water for 30 min, followed by drying under N_2_ flow. Prior to SECCM scanning, the entire surface of the prepared electrode (termed AuNP@HOPG, herein) was immersed in the electrolyte, and macroscopic electrochemical cleaning was performed in 0.1 M NaOH. This was conducted with a Pt counter electrode and a leakless Ag/AgCl reference electrode (LF-2-100, Innovative Instruments Inc., USA), and 10 cyclic voltammetric cycles were performed from −1.0 V to 0.75 V *vs.* AgCl with a voltammetric scan rate of 500 mV s^−1^.

Macroscopic measurement of drop cast AuNPs was conducted on a glassy carbon (GC) disk electrode (diameter = 3 mm). Preparation of the drop-cast AuNPs on this electrode followed the same procedure outlined above for the AuNP@HOPG sample. Macroscopic electrochemistry was recorded in a 3-electrode cell, using a Pt counter electrode and a leakless Ag/AgCl reference electrode, with 3 cyclic voltammetric cycles performed from −0.5 V to 0.7 V *vs.* Ag/AgCl at a scan rate of 100 mV s^−1^.

### 2.2 Preparation of SECCM probes

To prepare the micropipettes, borosilicate capillary tubes were used (BF100-50-10, Sutter Instruments, USA; dimensions: outer diameter, 1.0 mm; inner diameter, 0.5 mm; length 100 mm). These were placed in a two-stage capillary gravity-puller (PC-100, NARISHIGE Group, Japan) and pulled to a ∼1 μm tip diameter. For the first stage, the parameters were heat 63.3, weight 0, and slider 9. For the second stage, the parameters were heat 61.7, weight 0, and slider 4.5. The micropipette probe was then filled with 5 mM NaBH_4_ in 0.1 M NaOH electrolyte, with a small amount of silicone oil added to the top to prevent evaporation from the back of the probe over the course of a scan. The quasi-reference counter electrode (QRCE) used was an AgCl-coated Ag wire, which was prepared *via* the electrochemical oxidation of the surface of an Ag wire (Goodfellow, UK; thickness, 0.125 mm; purity, 99.99%) in a solution of saturated KCl. The open-current potential of this QRCE was measured and calibrated against a saturated calomel electrode (SCE) (CHI150, CH Instruments, Inc., USA), against which all potentials are reported. The QRCE is known to possess a stable potential on the timescale of several hours under SECCM conditions, as previously reported.^42^

### 2.3 SECCM setup

SECCM was carried out on a home-built scanning electrochemical probe microscope, based on a previously reported setup.^30,43–45^ This consisted of a 3-axis piezoelectric positioning stage (200 μm × 200 μm × 200 μm range, Nano-3D200, MadCityLabs, USA), with a micropipette attached such that the tip was positioned above the electrode region of interest. The location of the tip with respect to the surface was adjusted with a micropositioner (9064-XYZ-M, Newport, USA) prior to the experiment.

The AuNP@HOPG working electrode (WE) was attached to an SEM stub (SEM pin stubs, Microscopy Solutions Pty. Ltd., Australia) using conductive double-sided copper tape (AGG3397, Agar Scientific, UK) before being placed in a polypropylene container (HPL931, 100 mL, Lock&Lock, South Korea) which served as an atmosphere-controlled environmental cell, as previously reported.^39^ To this cell was attached a gas inlet port (Omnifit Connector, Kinesis, UK) which enabled the supply of N_2_ to the cell, and a circular hole of ∼4 mm in the top of the lid enabled the approach of the micropipette while maintaining a positive flow of gas through the cell. The N_2_ was passed through a bubbler containing deionized water in order to humidify the gas, before passing through a variable area flow meter (2510 2A12, Brooks Instrument, USA) to maintain constant supply (∼100 ccm), and eventually being delivered to the cell *via* PVC tubing. Two circular holes (∼2 cm) were drilled into the lid and replaced with cover glass (thickness: 0.13–0.17 mm, dimensions: 24 × 50 mm^2^) in order to supply the surface with light and provide an optical pathway to enable viewing of the micropipette tip and surface using an optical camera (Axiocam ERc 5s, ZEISS, Germany) fitted with a magnification lens (44 mm/3.00× InfiniStix, Infinity USA).

This entire setup was placed on an optical breadboard to which the positioner, environmental cell, and camera were mounted, and this breadboard was attached to a vibration isolation stage (25BM-8, Minus K Technology, USA). The vibration isolation stage with the above attachments was placed in a home-built aluminium Faraday cage lined internally with acoustic insulation foam (Adhesive PUR Foam, RS PRO, UK).

Before beginning the SECCM experiment, an electrical connection was made to the QRCE in the tip, and the SEM stub housing the AuNP@HOPG WE. This latter connection fed to a variable-gain low-noise current amplifier (DLPCA-200, FEMTO, Germany). The tip was then moved to the desired position before closing the Faraday cage and programming the experiment in LabVIEW (National Instruments, USA) with dedicated Warwick electrochemical scanning probe microscopy (WEC-SPM) software (http://www.warwick.ac.uk/electrochemistry/wecspm).

The SECCM experiment was conducted in the voltammetric hopping-mode with the tip being repeatedly approached to the surface in a grid pattern to obtain spatially-resolved electrochemical information.^46^ At each location, the tip was approached toward the surface at 2 μm s^−1^ with an applied potential (*E*_app_) on the QRCE of 0 V with respect to the ground. This was until the electrolyte meniscus protruding from the tip contacted the surface, inducing a double-layer charging current. When this charging current exceeded ±5 pA, the approach was stopped and the potential was stepped to −0.93 V *vs.* SCE at the QRCE before the CV measurement. It should be noted that since the potential was applied at the QRCE, not the surface, the surface is at a potential opposite to that of the QRCE (*E*_surf_ = −*E*_app_). Three CVs were taken (−0.93 to 0.68 V *vs.* SCE) at a voltammetric sweep rate of 2 V s^−1^. Finally, the tip was moved away from the surface before being moved to the next scan location (with a hopping distance of 5 μm), with the above process being repeated until a measurement was taken at the full grid of positions. Positioning of the probe was constantly monitored, and the *z*-piezo position at each scan location was used to generate a topographical map of the scan area.

During the course of the experiment, the current at the WE surface (*i*_surf_) was recorded every 4 μs and averaged 512 times, giving a data acquisition rate of (512 + 1) × 4 = 2052 μs per datapoint. The data acquisition was handled by an FPGA card (NI USB-7855R, National Instruments, USA), which sat between the LabVIEW interface and the SECCM instrument. Raw data was processed in the MATLAB environment (MathWorks, USA) and the Python environment (Python Software Foundation, USA), and graphed using SigmaPlot (Systat Software Inc., USA), OriginPro 9.0 (OriginLab, USA), and the Python Matplotlib library.

### 2.4 Analytical techniques

Optical microscopic imaging was performed on an Axiolab 5 microscope (ZEISS, Germany) with a range of fitted objectives (5×, 10×, 20×, and 50×). Images were collected *via* an attached Axiocam 105 color (ZEISS, Germany) optical camera. Post-scan co-location and imaging of AuNPs was performed *via* SEM on a Nova NanoSEM 450 FEGSEM (FEI, USA) and a Verios 5 XHR SEM (Thermo Scientific, USA). Images collected *via* SEM were analysed through the use of the open source software Fiji (ImageJ).

## 3 Results and discussion

### 3.1 Single-entity SECCM and the BOR

**Fig. 1:**
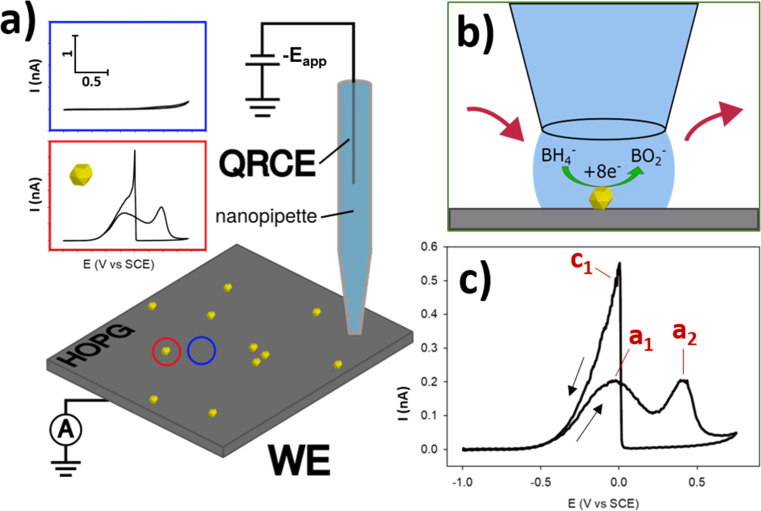
(a) Schematic of an SECCM experiment perfromed on an AuNP@HOPG sample. In the SECCM experiment, potential is applied at the QRCE (−*E*_app_) and the resulting current at the HOPG working electrode (WE) is measured. Blue inset shows typical CV of HOPG substrate, red inset shows typical CV of a single AuNP at the same current/potential scale. (b) Close-up of a single probe landing with an encapsulated NP performing the BOR. (c) A typical CV of a single AuNP on the HOPG surface with peaks labelled a_1_, a_2_, and c_1_. CV was collected during an SECCM experiment, from −0.93 to 0.68 V *vs.* SCE at a scan rate of 2 V s^−1^ in a solution of 5 mM NaBH_4_ + 100 mM NaOH.

The essential details of the SECCM experiment are provided in the Experimental section, but for ease-of-understanding, the general approach will be outlined here. Commercial AuNPs (∼200 nm diameter) were deposited *via* drop-casting onto the surface of freshly-exfoliated HOPG, and their electrochemical activity for the BOR was measured using SECCM. HOPG was selected as the ideal substrate for this study, due to its (electro)chemical inertness at BOR potentials, its cleanliness due to the preparation method, and also it is a suitable analogue for the kinds of carbon supports used in fuel cells.^47,48^ SECCM was operated in the voltammetric hopping mode,^46^ with an array of CVs generated (37 × 37 grid) under a N_2_ atmosphere (Fig. 1a). Using this method, individual NPs (and small NPCs) were able to be encapsulated individually, in order to measure the electrochemistry of the single-entity (Fig. 1b), rather than measuring the multitude of NPs on the functionalised surface simultaneously *via* traditional, macroscopic measurements. This allowed the interrogation of the activity variation amongst otherwise similar-looking NPs, as well as a determination of the effects of cluster size in NPCs.

The BOR (conducted with 5 mM NaBH_4_ and 0.1 M NaOH supporting electrolyte) is a complex, multi-step reaction. This reaction was chosen as a model case due to its relevance to potential fuel-cell applications, as well as the 8 electrons which are passed for the full conversion of BH_4_^−^ to BO_2_^−^, generating relatively high currents on a per-molecule basis.^15^ Before exploring the expanse of electrochemical information uncovered by this technique, it is useful to outline the basic, contemporary understanding of this reaction and it's voltammetric characteristics. In basic conditions, BH_4_^−^ can undergo full oxidative conversion to BO_2_^−^*via* the reaction outlined in eqn (1).^49,50^ However, BH_4_^−^ also undergoes spontaneous hydrolysis, to form BH_3_OH^−^, as per eqn (2).^49^ This species can also undergo electro-oxidation to form BO_2_^−^, and is believed to be a reaction intermediate of the BOR, providing an indirect (and not desired) pathway for the formation of BO_2_^−^.^51^ The indirect reaction has multiple pathways, with some resulting in H_2_ production, drawing parasitic current and potentially altering the reaction conditions.^51^ Optimisation of this reaction for DBFC applications has focused on designing catalytic materials which prioritise the direct pathway, and limit the indirect pathway.

1 BH_4_^−^ + 8OH^−^ → BO_2_^−^ + 6H_2_O + 8e^−^

2 BH_4_^−^ + H_2_O → BH_3_OH^−^ + H_2_

When investigating the CV of BOR on Au the first peak during the anodic (towards positive potentials) sweep at ∼0 V *vs.* SCE, here termed a_1_ is conventionally understood to be due to the direct oxidation pathway, and the second anodic peak at ∼0.4 V *vs.* SCE, a_2_, is due to the indirect pathway (Fig. 1c).^51^ This is followed by the electro-oxidation of the AuNP surface at ∼0.45 V *vs.* SCE, which switches off the reaction, as the Au oxide surface is catalytically inert for the BOR. In the cathodic (towards negative potentials) sweep a single peak is observed at ∼0 V *vs.* SCE, which is the “switch-on” peak resulting from the reduction of the Au surface oxide, and subsequent BOR activity. Previous research has suggested that this peak is a complex mix of catalytic processes arising from the oxidation of BH_4_^−^ and any number of intermediates including BH_3_OH, BH_2_(OH)_2_, and BH(OH)_3_, which are adsorbed to the AuNP surface.^15,51^

**Fig. 2:**
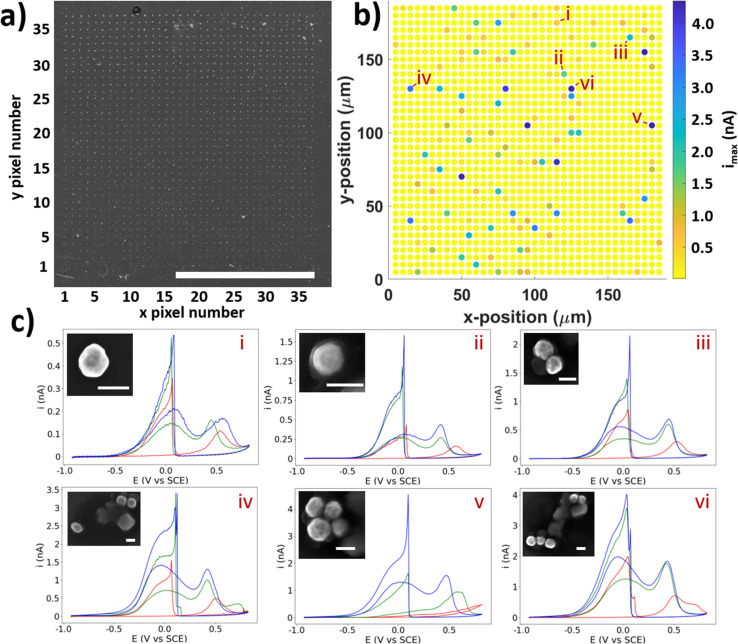
(a) SEM image of a scan area on AuNP@HOPG after SECCM experiment, showing probe contact *x*–*y* locations labelled 1–37. Scale bar = 100 μm. (b) Electrochemical image of BOR activity on AuNP@HOPG showing peak current (*i*_max_). Selected locations are labelled *i*–*vi*. (c) CVs from selected NPs labelled *i*–*vi* in order of increasing #NPs (1–5). CVs are three cycles in the order red, green, blue. Scale bar = 200 nm. Note the changing *y*-axis as *i*_max_ increases from *i*–*vi*.

After the SECCM experiment, a grid of probe-landing locations can be observed in SEM (Fig. 2a), allowing for post-experiment colocation of the electrochemical data with the regions encapsulated by the probe droplet. These locations are visible due to the residue left behind by any electrolyte left after droplet detachment. This effectively allows any locations of higher electrochemical activity to be imaged in SEM, and any NPs found in the residues can be paired with their associated electrochemical data (CVs).

The SECCM results give a spatial mapping of the electrochemical information on the surface (Fig. 2b). A CV with 3 cycles was collected at each landing site, the collation of which allows the information to be displayed as an electrochemical movie, where current is played back as a function of *xy* position and potential (as seen in the ESI, movie S1†). The map of peak current (*i*_max_) is shown in Fig. 2b. Most of the electrochemical data collected from this experiment is simply HOPG (*i.e.* no NP was contacted), as evidenced by the yellow dots in the SECCM map. Amongst this ‘background’ data of the substrate, spots with higher activity can be seen, corresponding to locations which contain NPs, whilst many of the residues which are known to contain NPs (Fig. 2a.) are not visible in this map (*i.e.* they are indistinguishable from the HOPG background). This apparent inactivity for the BOR of many particle-containing locations will be explored further below. Of the total locations, there were 178 single NPs, 49 NPCs of size 2, 28 NPCs of size 3, 10 NPCs of size 4, and 3 NPCs of size 5. A table containing these totals is included in the ESI, Table S2.1.† This is a considerable amount, and is a clear demonstration of the ability of SECCM to generate large datasets of both single entities and clusters to a degree which has not been demonstrated yet. A statistical treatment of this dataset follows later in this report.

### 3.1 General findings

First it is important to characterise the complex AuNP-functionalised surface. The distribution of drop cast particles was investigated *via* SEM imaging at a magnification of 10 000× with a series of 10 images. The spatial density was found to be 28 500 NPs per mm^2^ and the average nearest neighbour distance (NND) was found to be 2.26 μm, with moderate variance, indicating some clustering. The full results and associated statistics are presented in the ESI, Table S1.1–2.† Furthermore, a selection of 50 NPs was more closely investigated *via* SEM at a magnification of 5 000 00×. A variety of NP shapes was observed, some with a higher degree of faceting, and others with no discernible crystallographic facets. The average diameter of the NPs was 205 nm, and the diameter distribution was found to be quasi-normal, ranging from ∼150 to ∼250 nm at the extremes (ESI, Fig. S1.3†).

From the collection of particles which showed activity for the BOR in the SECCM experiment, a number have been selected to be highlighted as examples of “typical” CVs for the BOR on AuNPs (Fig. 2c, labelled *i–vi*). These are in increasing order of #NPs, and it can be seen that the current is clearly proportional to the number of NPs (*vide infra*). The first cycle of the CV (Fig. 2c, shown in red) consistently shows no a_1_ peak, but does show an a_2_ peak. As alluded to above, this a_1_ peak is due to the direct BOR, which cannot proceed if there is surface blocking by absorbed intermediates formed by the spontaneous hydrolysis of BH_4_^−^ (eqn (2)).^52^ It is proposed that the presence of a shifted a_2_ peak at approximately 0.5 V *vs.* SCE is due to the removal of absorbed species on the AuNP, and oxidation of the surface, which subsequently becomes active for the BOR on further cycling of the potential.^52–54^

In all cases, the successive cycles (in red, green, and blue) show a gradual increase in BOR activity, as the AuNP surface continues to undergo gradual electrochemical cleaning and/or surface restructuring due to the formation/stripping of oxide layers.^55^ This is crucial for those wishing to study single entities, as these particles already underwent 10 cycles of electrochemical cleaning (*via* macroscale electrochemistry, see ESI, S1.2†), yet still do not display a steady (*i.e.* unchanging) current with potential cycling. This indicates that comprehensive pre-treatment of catalytic NPs is required to measure intrinsic activity, or that absorbed surface species, such as capping ligands used in NP synthesis, must be removed or replaced before use for catalysis. In the least, care must be taken to prevent restructuring of the NP surface if a stable current is desired. For this reason, the effect of oxidative switching potential on the electrochemical stability of single entities is suggested as further study of electrocatalytic NPs of any kind.

Across the range of CVs, *i*–*vi* in Fig. 2c, some major differences can be seen which are not simply due to differences in electrocatalytic activity. In particular, some CVs have different shapes and peak ratios, which do not obviously correspond to superficial morphological differences (from SEM, see Fig. 2c insets). For example, the particles labelled *i* and *ii* in Fig. 2c are both single NP locations, yet the “switch-on” peak c_1_ for *ii* displays a 3-fold higher current (*i* = 0.54 nA, *ii* = 1.58 nA). Despite this, the a_1_ and a_2_ peaks only display a marginally higher current, with *i* displaying values of 0.21 nA and 0.17 nA, and *ii* displaying values of 0.31 nA and 0.43 nA, for a_1_ and a_2_, respectively (all for the 3rd cycle). This is a result which cannot be explained by size, as *ii* is in fact the smaller of the two particles (*i* ≈ 200 nm, *ii* ≈ 180 nm diameters). The shape of the c_1_ peak is also different between these particles, with the NP labelled *ii* showing an obvious shoulder on the negative side of the peak, which is also visible in other CVs in this study, to differing degrees (such as in Fig. 2c NPs *iii*, *iv*, and *v*). The a_1_ : a_2_ peak ratios also appear different for NPs *i* (1.23 : 1) and *ii* (1 : 1.38), indicating that particle *ii* is more active for the indirect pathway than *i*, even when normalising for direct-BOR activity.

These results qualitatively demonstrate the large variance of activities displayed by otherwise similar-looking particles, with a high degree of variation in peak size, shape, and ratio. This is a crucial finding, which has been explored by prior SECCM studies: that visibly-similar particles display considerably varied electrochemical activities for a wide array of catalytic processes, which conventional macroscopic electrochemistry will fail to uncover. SECCM, among other techniques, is thus required to probe this variance, and by this method any feature of the CV can have its variance investigated statistically, enabled by the considerable dataset generated by SECCM. A quantitative analysis of this is upcoming in this report for a selection of informative CV parameters including onset potentials, peak currents, and total charge passed during the forward/reverse sweep, *etc.*

It should however be noted that SEM images of similar particles may appear different depending on the nature of the surrounding residue. This residue is formed by evaporation of the electrolyte left on the surface after the probe has detached. Some particles lie just outside of this residue (yet were still encapsulated by the droplet, as evidenced by the electrochemical response), as is the case for the particle labelled *i*, while others are buried in the residue such as particle *ii*. The residue often prevents clear investigation of the NP shape and surface structure, as the residue will “burn” under high-energy electrons, leading to the cloudy image of particle *ii*. In fact, many particles which are buried in the residue are not visible by secondary-electron (SE) imaging, and are only visible *via* back-scatter electrons (BSE) imaging, which is sensitive to atomic mass. Attempts were made to remove the residues post-experiment (*i.e.* by bathing in a weak acid/base), in order to uncover these particles, with little result. This effectively prevents an in-depth, identical-location development of structure–activity relationship for any particle which is concealed by a residue. Attempting this experiment in reverse is possible (SEM followed by SECCM), but challenging due to the inability to target specific NPs in conventional SECCM (although it is possible under some conditions with optically-targeted SECCM),^56^ and potential degradation of the NPs under the high-energy electron beam.

**Fig. 3:**
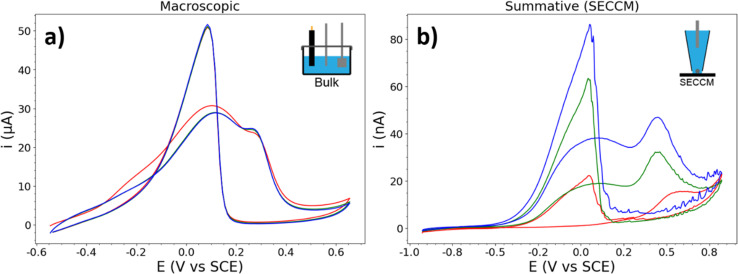
CVs of BOR on AuNPs from (a) a macroscopic experiment. CVs were recorded in a 3-electrode cell, using a Pt counter electrode and a leakless Ag/AgCl reference electrode, with 3 CV cycles performed from −0.5 V to 0.7 V *vs.* SCE at a scan rate of 100 mV s^−1^. (b) A summation of the currents of all AuNPs investigated in the SECCM experiment (scan rate of 2 V s^−1^). In both CVs the cycles are shown in the order: red, green, and blue (cycles 1, 2, and 3).

It is helpful to compare these “typical” CVs to that which is measured during conventional macroscopic experiments. An AuNP-functionalised glassy carbon (GC) macrodisk electrode (3 mm diameter) was prepared following the exact procedure used for the HOPG-supported AuNPs (including electrochemical cleaning), resulting in a CV displaying the activity of the total population of NPs present on the surface (Fig. 3). The comparison between GC and HOPG is possible due to the similar chemical makeup, surface functional groups, and (electro)chemical stability.^57,58^ This CV displays a BOR current which is more constant per cycle, as expected for the BOR on a large number of NPs, and the a_1_ and a_2_ peaks are broader and less separated, likely due to the subtle variation of peak potentials on different NPs that make up the ensemble. This is also visible, albeit to a lesser extent, in the CV which results from summing up the currents of all of the NPs measured with SECCM (Fig. 3b). The c_1_ peak is also less abrupt (more gradually sloped) in both the macroscopic and summative CVs, compared to the single NP CVs presented herein, due to the distribution of c_1_ peak potentials amongst the different NPs. Key differences still exist between the macroscopic and summative-SECCM CVs, notably the growth of the current response, and the difference in peak ratios.

To further this comparison with the SECCM experiment (which was conducted at 2 V s^−1^), the AuNP-functionalised (GC) electrode was also measured at 2 V s^−1^ (see ESI, S2.2†). This had a minor effect on peak shape and position, reflective of the differences between the CVs in Fig. 3. The a_2_ peak is shifted positive, and is more defined, in the faster scan rate CV. The comparison of the two CVs in Fig. 3 with the single-NP CVs presented herein demonstrates the importance of single-NP measurements, as the natural variation of NP electrocatalytic activities are effectively “washed-out” by macroscopic measurements.

### 3.2 Active/inactive NP locations

A standout result of this work is the surprising number of particle locations which did not show BOR activity. For single NP locations, 120 of the 178 particles (67.4%) displayed CVs which were indistinguishable from that of the surrounding HOPG, despite these NPs being clearly located within the residues (*via* SEM imaging), implying that they were within the SECCM meniscus during measurement. This key finding is one which has not been reported before, as previous SECCM experimentation has focused on finding electroactive NPs *via* their current–voltage response, followed by post-experimentation imaging. By reversing this process (*i.e.* locating the NPs in SEM, followed by investigating their electroactivity) it has been found that many of the particles which were encapsulated did not display any apparent current response above the HOPG support. This is a significant result, as a standard drop-casting procedure was followed, with a freshly exfoliated HOPG surface. Typical macroscopic experiments assume that the amount of material deposited onto the surface is the amount of active material for the electrochemical process of interest. This finding shows this assumption to be potentially untrustworthy, and may lead to overestimation of the actual electroactive material, potentially leading to a perceived decrease of the intrinsic activity of the particles.

There were also a significant proportion of NPs which displayed “abnormal” activity. These CVs showed clear BOR activity, but did not show the typical a_1_, a_2_, and c_1_ peaks, and so the characteristic parameters used in the statistical analysis of BOR on AuNP@HOPG could not include these particles. As opposed to the “inactive” NP locations, the proportion of abnormal locations does not decrease as a function of cluster size, suggesting that the cause of the abnormal activity of these particles is different than the cause of particle inactivity. A qualitative examination of the electrochemistry of a selection of these particles is featured later in this report.

It was hypothesised that the spatial location of a NP location in the scan area could be related to the “type” of nanoparticle activity (*i.e.* that abnormal or inactive particles could be spatially clustered). This was based on the idea that particular areas of the support may have had impurities which prevented sufficient particle-to-support contact. In order to rule this out, a spatial autocorrelative index (Moran's I) was calculated for each “type” of NP location, as well as for all NP locations and compared to Monte Carlo simulations of the same size dataset (see ESI, S3.1†). It was determined that the NP locations of each type were within the 95% confidence interval for the expected results, indicating that the NP locations were effectively random across the scan area (*i.e.* no “clumping” of locations). To further investigate this, the nearest-neighbour-distance (NND) was calculated for each type of location, and compared to Monte Carlo simulations of the expected NND (see ESI, S3.2†). It was found that for all NP locations, the observed NND was greater than the mean, and was outside the range of expected values (assuming all locations correspond to a single Au NP). This was expected, as the NP locations should have higher NNDs than randomness would predict due to low-NND NPs being treated as single clusters (*i.e.* NPCs were treated as single NP locations), and the co-deposition of multiple NPs. However, this was not observed for the abnormal NP locations, and the observed NND was within the range of expected values. This indicates that the subset of all NP locations which are classified as abnormal is a randomly distributed subset of all NP locations. This supports the claim that abnormal NP locations do not arise from the same origin as the inactive locations.

The dataset explored herein does not allow the determination of the origin of abnormality or inactivity of SECCM locations. However, through in-depth exploration of the dataset, certain hypotheses (shown schematically in the ESI, Fig. S2.1†) can be tested, of which some are explored here. Firstly, the particle may detach from the substrate upon probe contact (*i.e.* due to electrostatic repulsion caused by the establishment of the electrical double layer), and be left in the residue (*i.e.* re-deposited) during probe detachment (Fig. S2.1a†). This is unlikely, considering the electrochemical pre-treatment protocol used during sample preparation, which is expected to irreversibly remove any loosely adhered particles, prior to SECCM.

**Fig. 4:**
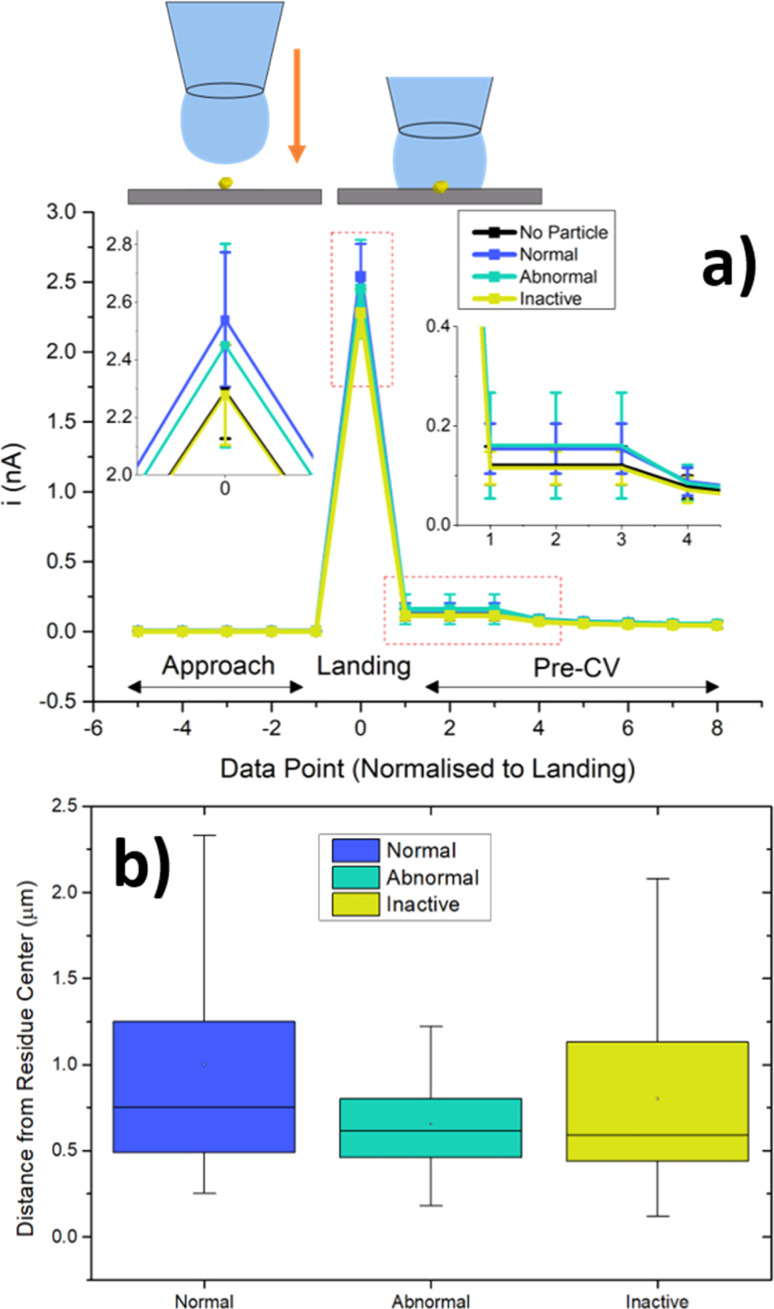
(a) Current values from the SECCM probe approach, landing, and pre-CV regions of the dataset, averaged for each classification of probe landing. Error bars show a standard deviation from the mean at each entry. Insets show a close up of the landing current spike (left inset) and a close up of the pre-CV transient (right inset). Note that each datapoint is separated by a time of 2052 μs. The applied potential during approach (*i.e. x*-axis < 0) was 0.07 V *vs.* SCE, which switched to −0.93 V *vs.* SCE after landing (*i.e. x*-axis ≥ 0), giving rise to the characteristic current transient shown. (b) Box plot of radial distance of single AuNPs in their SECCM landing residue, for each classification of particle.

An analysis of the probe approach transient has revealed noticeable differences between the “landing” (probe contact) and “pre-CV” current values and distribution for locations with normal BOR response, and those with no BOR response (Fig. 4a). This provides a diagnostic signal which confirms whether a particle was contacted before the CV. Importantly, inactive locations do not show this diagnostic signal, meaning there is no evidence that a particle was contacted by the droplet, whilst abnormal locations show landing transients indicative of particle contact. This indicates that the “inactive” particles truly showed no activity for the duration of meniscus contact. In other words, it is unlikely that the cause of perceived inactivity arises from the particles being physically disconnected upon probe landing, *e.g.* due to the spontaneous formation of hydrogen bubbles (see eqn (2)).

In order to rule out the possibility that “inactive” particles were simply not encapsulated in the droplet during measurement, the SEM image of the scan was analysed, and the radial distances (from the centre of the residue) was measured for each classification of single NP (Fig. 4b). The normal and inactive locations contained particles with similar distances and distributions, ruling out this possibility, confirming that “inactive” particles were encapsulated by the SECCM meniscus cell to the same extent as normal ones. This is in agreement with previous single-particle SECCM studies, where it was demonstrated that the location of a particle within the probe droplet during measurement has little impact on the electrochemical response.^59^ Interestingly, the abnormal locations contained particles which were significantly closer to the centre, with a narrower distribution.

Alternative hypotheses remain, and are considered here. The particle may be passivated (or the reaction altered) by adsorbates (Fig. S2.1b†). The obvious candidate here is the capping agent, citrate, although the washing and pre-treatment steps make this unlikely, due to the high solubility of citrate in water. Some particles may be passivated by deposits of citrate formed during the drying process: a sufficient deposit of citric acid could alter the local pH enough to hydrolyse the BH_4_^−^, however this is unlikely to lead to the inactive CVs shown here as the hydrolysis product is still electrocatalytically active. Another candidate for NP passivation is corrosion product from the carbon support, which has been previously demonstrated to have a poisoning effect on metal NPs undergoing a structure-sensitive reduction reaction (*e.g.* the oxygen reduction reaction).^60^

The particle may be electrically disconnected from the carbon surface by the citrate ligands, or other impurities in the drop casting medium (Fig. S2.1c†). This is also an unlikely cause, since electron tunnelling has been reported across a few nm of organic material.^61^ This may be an explanation for those abnormal CVs that appear to be affected by ohmic resistance, but complete deactivation of the particle would require a sufficiently thick insulating barrier. A further hypothesis may be that the location of the NP relative to the structural features of the HOPG support (*i.e.* NPs adhered at step edges and imperfections) may have an impact on NP activity. A previous study has shown that the step edge response cannot be isolated from the basal plane response at ZYA-grade HOPG at the length scale employed in this experiment,^62,63^ however it is not yet clear how this could lead to particle inactivity.

This leaves the hypothesis that certain particles are intrinsically inactive for the BOR, due to structure or morphology (Fig. S2.1d†). A deeper investigation of these hypothetical deactivation pathways is warranted, and requires a more in-depth correlation of structure and activity. This is possible with SECCM and SEM, but requires more well-defined structures: the use of highly monodisperse, shape-controlled particles allows a direct correlation to be formed between the structure of a particle and its catalytic performance. This presents an attractive target for further SECCM experimentation.

### 3.3 Cluster size effects

**Fig. 5:**
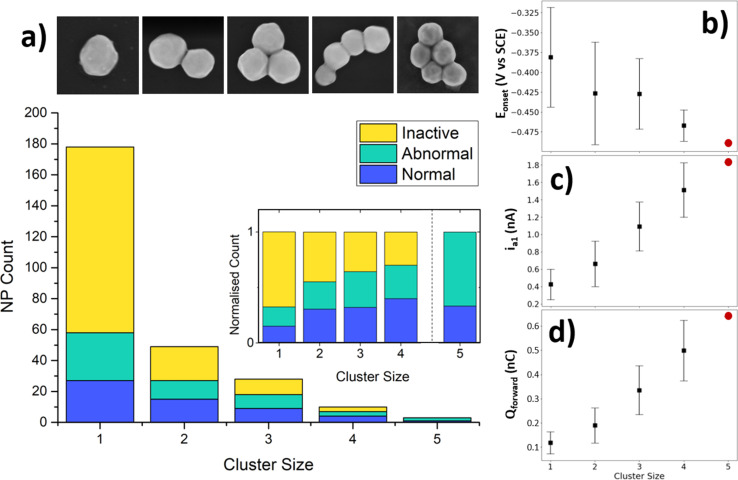
Population analysis and effect of cluster size on electrochemical features of the BOR (a) population of locations for each cluster size, with colour-coded classification of electrochemical response. Inset shows these populations normalised. Cluster size = 5 is included illustratively, as the population (*n* = 3) is too low for inclusion in the statistical analysis. Examples of AuNP clusters in SEM are given above. Selected electrochemical features of AuNPs classified as normal as a function of cluster size for (b) *E*_onset_ (V *vs.* SCE), (c) *i*_a1_ (nA) and (d) *Q*_forward_ (nC). Note: the data from cluster size = 5 for normal particles contains only one data point (red circles), and so is only included in order to demonstrate the predictive ability of the statistical trend.

During the course of the SECCM experiment, the probe encountered a range of NP cluster sizes, from single-NPs to (in this work) clusters of size = 5. Naturally, the number of clusters encountered decreased as a function of cluster size (Fig. 5a), as they are rarer than single-NPs on the electrode studied. Interestingly, the ratio of normal, abnormal, and inactive NPs changes as a function of cluster size (Fig. 5a inset). The number of inactive locations decreases relatively with cluster size, indicating that particle inactivity tends to be a single-NP phenomenon, rather than a whole-cluster phenomenon. Alternatively, the relative number of abnormal and active locations tends to increase with cluster size.

An important factor which can have an effect on the catalytic activity of NP clusters is the interparticle distance.^64^ To determine whether the impact of this is measurable in our results, the interparticle distances were measured at locations with cluster size = 2, grouped into active, inactive, and abnormal locations, and an analysis of variance was conducted (see ESI, S3.3†). The majority of the locations contained clusters with interparticle distance = 0 (*i.e.* 80–90% of clusters were in physical contact) resulting in a median and mode of 0 for each group. The analysis of variance results showed that the population means and variances were not significantly different at the 0.05 level, indicating that no impact of interparticle distance could be measured.

As outlined earlier, the large detested generated by the SECCM experiment is sufficient for analysis of the wide variance of activities amongst the NP locations. Previous SECCM experimentation on single-NPs has tended to highlight a few cases of single-NP activity from within the dataset,^33,36^ but this often fails to demonstrate the wide variance of activities, and the large datasets such studies can achieve. The goal herein is to exploit the whole dataset, and explore all of its findings. To achieve this, a selection of indicative characteristics of the BOR CV is shown here for both single NPs, as well as how the activity distributions change as a function of cluster size, with all others presented in the SI (see ESI, S3.4†). Those shown here are onset potential of the BOR (*E*_onset_), a_1_ peak current (*i*_a1_), and forward charge passed (*Q*_forward_).

The *E*_onset_ is defined as the potential which the BOR current of the a_1_ peak reaches 4% of the maximum current (*e.g.* associated with either a_1_ or a_2_) on the forward sweep. *E*_onset_ is taken to be a heuristic of the BOR kinetics of the NP, as those NPs which achieve this percentage sooner will require a lower overpotential. For the single NPs, this has a wide distribution of values, from −0.50 V to −0.26 V *vs.* SCE, with a mean of −0.38 V *vs.* SCE (for the 3rd cycle). This indicates that the intrinsic activity of single NPs has a significant variance, as indicated by the relatively large standard deviation (Fig. 5b, scale bars are 1 standard deviation). As the cluster size increases, the *E*_onset_ for the BOR becomes more negative (*i.e.* an earlier onset), meaning that the cluster achieves 4% of maximum current sooner when more particles are present. The distribution also decreases as a function of cluster size, which should be expected as the *E*_onset_ will be an average of multiple particles.

The peak BOR current at a_1_ for single NPs takes values from 0.12 nA to 0.62 nA at 0.05 V *vs.* SCE, with a mean of 0.37 nA as seen in Fig. 5c. This value is taken to be a measure of the peak capacity of the NP to drive the direct BOR, with its variation most likely due to variations in the active surface area (*e.g.* particle size). The NPs are only approximately ∼200 nm as supplied, and further studies can probe the effect of increasing NP size on these CV parameters, in order to determine if any of the variance is unexplainable by this factor alone. Naturally, the *i*_a1_ increases linearly as a function of cluster size, whilst retaining its interparticle variance.

The NP cluster size should also have an effect on the total charge passed (*Q*_forward_), which can be seen scaling semi-linearly in Fig. 5d. The *Q*_forward_ indicates the sum of all processes, both direct and indirect BOR, before the particle is “switched off” by the surface oxidation. The *Q*_forward_ values for a single NP range from 0.05 nC to 0.21 nC, with a mean of 0.11 nC. This is a 4-fold spread in amounts of charge passed, which is clearly not simply a result of NP size, and thus reflects some aspect of intrinsic NP activity for the BOR. Additionally, the distribution of the *i*_a1_ stays relatively steady as a function of cluster size, with only a minor increase in variance, while the distribution in *Q*_forward_ increases noticeably. One might expect the distribution of *Q*_forward_ to narrow as more particles are included, as the effect of the variance of NP size will be smoothed out due to taking the sum of multiple particles. However, the presence of an unknown number of BOR-inactive NPs will produce a wider range of possible *Q*_forward_ values depending on the number of such particles (which would not be expected for *E*_onset_).

### 3.4 Correlation analyses

**Fig. 6:**
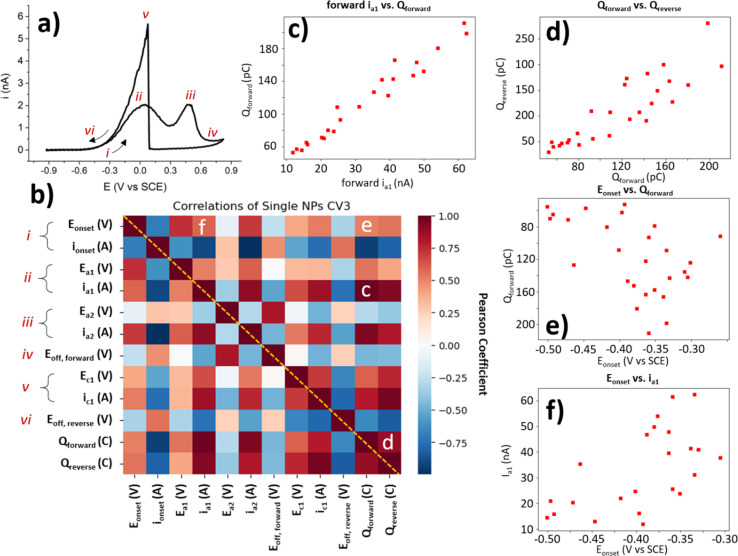
Correlation analysis of main electrochemical features in the CV of BOR on AuNP@HOPG. (a) Example CV of a single NP with labels of CV features used in correlation analysis. (b) Correlation heatmap of Pearson coefficient for each of the main features labelled in (a). These features have more in-depth definitions in the ESI (Table S3.1†). Selected correlations are labelled c-e. Correlation plots of (c) *i*_a1_ (nA) *vs. Q*_forward_ (pC), (d) *Q*_forward_*vs. Q*_reverse_ (pC), (e) *E*_onset_ (V) *vs. Q*_forward_ (pC), and (f) *E*_onset_ (V) *vs. i*_a1_ (nA).

The collection of a significant number of BOR-active NP locations enables further statistical correlation of the CV parameters. As each of these parameters reveals important information about the NP activity for the BOR, each will be explained briefly in turn. The “*E*_onset_” discussed earlier is the potential at which the current reaches 4% of the maximum current, and “*i*_onset_” is the current at this potential (Fig. 6a label *i*). The a_1_ and a_2_ peak potentials (*E*_a1_ and *E*_a2_) and currents (*i*_a1_ and *i*_a2_) are included here also (Fig. 6a labels *ii* and *iii*). The “*E*_off, forward_” is the potential at which the local minimum current is reached, after the BOR switches off on the anodic sweep (Fig. 6a label *iv*). Also included is the c_1_ peak potential and current (*E*_c1_ and *i*_c1_, Fig. 6a label *v*). The final labelled feature is the “*E*_off, reverse_” which is the potential at which the current returns to the *i*_onset_ value on the reverse sweep (Fig. 6a label *vi*). The forward charge (*Q*_forward_) and the reverse charge (*Q*_reverse_) are simply the area under the CV for the forward and reverse voltammetric sweep, respectively.

Since each NP location with a “normal” BOR CV provides a value for each of these parameters, it is possible to conduct an exploratory statistical analysis to uncover which parameters are highly correlated, and which show a low correlation. This is done here by generating a heatmap of Pearson correlation coefficients (Fig. 6b). A Pearson coefficient close to 1 indicates a high positive correlation (dark red squares in the heat map), and values close to −1 indicate a high negative correlation (dark blue squares). All of the parameters used to generate this heatmap are only collected from the 3rd CV cycle for the single NP locations classified as “normal”, as these are the only BOR CVs which provide values for all of these parameters.

A selection of these correlations is plotted alongside the heatmap (Fig. 6c–f). These figures (as well as the coefficients they correspond to) have had outliers removed (using the Mahalanobis distance with threshold = 3). Some parameters are expected to display meaningful correlations, such as the *i*_a1_ and the *Q*_forward_ (Fig. 6c) with a Pearson coefficient = 0.96. This is expected, as a more BOR-active NP should have both a higher peak *i*_a1_ and pass more charge during the BOR. Another expected correlation, is that of the *Q*_forward_*vs. Q*_reverse_ (Pearson coefficient = 0.89). This is slightly less correlated than expected, as the activity of the NP for the BOR should correlate with both *Q*_forward_ and *Q*_reverse_. The final correlation highlighted herein is the *E*_onset_, which is expected to be a heuristic of NP activity. When correlated with the previously investigated parameters, *i*_a1_ current and Q_forward_, only minor correlation is found (Pearson coefficients = 0.58 and 0.61, respectively). In fact, the *E*_onset_ appears to be more highly correlated to *i*_a2_ (Pearson coefficient = 0.74) than to *i*_a1_. This demonstrates that the choice of heuristic for NP activity should be carefully selected when developing structure–activity relationships.

Heatmaps of this kind not only highlight the parameters which are highly correlated, but also show those parameters where a correlation might be expected, but not found. In this case, a mild positive correlation was found between the reverse peak position, *E*_c1_, and the first forward peak, *E*_a1_ (Pearson coefficient = 0.33). This is somewhat expected, as the *E*_c1_ peak indicates the potential of oxide stripping of the particle, which would be expected to correlate with the activity of the particle (*e.g. via* surface energy). However, no correlation was found between *E*_c1_ and the second forward peak, *E*_a2_ (Pearson coefficient = −0.06). This is somewhat surprising, as it seems to indicate that the potential of the a_2_ peak is not dependent on the factors involved in surface oxide stripping, such as surface energy, which potentially provides insight into the processes underway at a_2_. It is exactly this kind of surprising insight, provided by exploratory analysis of the massive dataset generated by SECCM, which this paper seeks to highlight.

To demonstrate the substantial capability of SECCM to conduct extensive exploratory statistical analyses, Pearson coefficient heatmaps are provided for the 1st, 2nd, and 3rd CV cycles, for the range of cluster sizes (ESI, S3.5–17,† cluster size = 5 omitted due to low number of locations). These heatmaps can be compared to visualise how these parameter correlations change as a function of either CV cycle or cluster size. Further experimentation should seek to maximise the value of the unique ability of SECCM to generate such large, statistically significant datasets by conducting targeted experiments which sufficiently control for structural features of interest. This allows for not just further exploration of the wide variance among similar NPs, but for explanatory structure–function relationships to be developed, by correlating this with the variance found in the structure of the NPs.

### 3.5 Outlying and “abnormal” electrochemistry

Here will be presented a brief, qualitative overview of some of the unexpected CVs collected in this experiment. If a NP location displayed BOR activity (*i.e.* displaying heightened activity which cannot be explained by HOPG alone), but does not resemble the expected BOR CV shape, it has been classified as “abnormal” and left out of the statistical analysis. However, due to the percentage of NPs which display such activity, they should not be ignored.

**Fig. 7:**
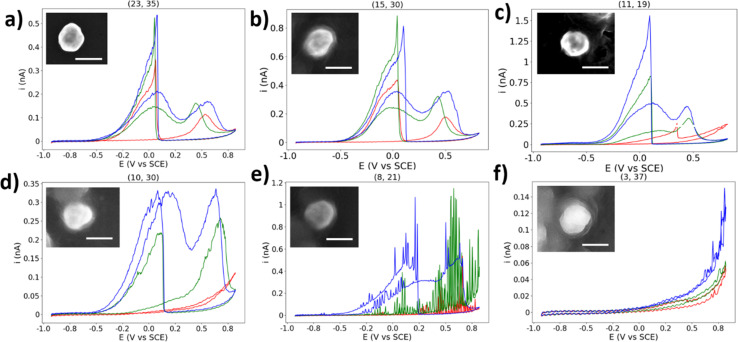
(a)–(f) Selected single AuNP CVs with normal/abnormal behaviour. CVs are three cycles in the order red, green, blue. Insets show SEM image of particle. Scale bar = 200 nm.

First presented is a NP classified as “normal” for comparison, due to the presence of well-defined a_1_ and a_2_ peaks in the anodic sweep, and a well-defined c_1_ peak in the cathodic sweep (Fig. 7a). In this CV, the c_1_ peak occurs at ∼0.1 V *vs.* SCE, which is normal for the majority of particle locations featured in this study. However, many particles displayed unintuitive CVs such as what is shown in Fig. 7b, with the potential of the c_1_ process changing per CV cycle. The potential of the c_1_ peak first appears at 0.04 V in the first CV, then 0.1 V in the second CV, and back to 0.04 V in the third CV (all *vs.* SCE). The c_1_ peak potential appears to shift back and forth in a manner not expected of a catalytic process. This position of this peak is due to the surface oxide stripping of the particle, and the processes underway have previously been suggested to be the oxidation of BH_4_^−^ and its oxidised forms: BH_2_(OH)_2_ and BH(OH)_3_.^51^ The unknown nature of the processes underway at the c_1_ potential makes a clear statement of the origin of this behaviour challenging.

The encapsulated particle does not always display BOR current on the first cycle, as shown in Fig. 7c. The simplest explanation for this is that the particle was at first not in contact with the droplet, which eventually becomes encapsulated during the measurement, leading to the current spike at 0.35 V *vs.* SCE. However, this would not explain why the non-BOR “background” current is so high for the first cycle (*e.g.* compared to naked HOPG), and returns to the expected value on subsequent cycles. Many particles display similar behaviours, and some particles show this in reverse, with the particle appearing to “switch-off” for the BOR.

The CV shown in Fig. 7d is an example of an abnormal CV which displays some similarity to the typical BOR CV, yet is not representative of the expected shape. This type of response is not an anomaly, however, as 14 of the 31 (45%) of the single NPs which are classified as “abnormal” display a qualitatively similar shape. The first CV shows no activity for the BOR, the second CV resembles that of a typical first CV, and the third CV resembles a typical CV with a c_1_ peak with half the expected current. It is unclear why this particle should show such low activity for the c_1_ peak, while eventually attaining activity for the a_1_ and a_2_ peaks.

The next abnormal CV is shown in Fig. 7e, which shows a CV similar to Fig. 7d, but with an apparent oscillation (“spiking”) in the measured current. This “spiking” in the current is suggested to be due to a NP oscillating on the surface of the substrate, repeatedly making and breaking electrical connection. This has been proposed previously in NP landing experiments.^65^ This may be expected here, as a build-up of charge on both the NP and substrate will result in repulsive forces which will dislodge an improperly adhered particle, which may explain why the frequency/magnitude of the oscillation increases significantly as the potential moves away from the potential of zero charge.^66,67^ This further supports the above explanation for the origin of the abnormal result found in Fig. 7d.

The final abnormal CV highlighted here is shown in Fig. 7f. This CV appears to show an activity similar to HOPG at first, but in the third cycle it grows to a current not expected for just a HOPG surface. It is suggested that this is a rare combination of an inactive particle location and an HOPG step-edge, which will have a higher activity at this potential than basal plane HOPG, due to a higher surface energy and more available surface area.^68^ The droplet appears to contact the step-edge on the third cycle, and many other locations show this “enhanced background” current at locations with BOR-inactive NPs, which can easily be confused with enhanced NP activity.

Other types of abnormal CVs include those with a clear c_1_ peak but no clear anodic peaks, those where the anodic peaks are shifted positive and appear resistive, and those which show normal BOR activity before becoming inactive. In this study, an attempt has been made to separate those results which appear typical of the BOR from those which are unexpected. However, this assortment of unexpected electrochemical results should make clear that this is not a simple process which can be achieved with straightforward qualitative methods. The true challenge with single-entity research is to differentiate between those “abnormal” outliers which are informative, and those which are merely by-products of the nature of the experiment. In any case, abnormal single-entities should not be discarded from any analysis as mere artefacts, and should be treated as real data until their elimination can be justified.

## 4 Conclusion

In this study, single Au NPs and clusters of Au NPs, of a range of sizes, were encapsulated within a droplet on the end of an SECCM probe, and CVs with 3 cycles were measured at each location. This produced a dataset of a significant size (*N* = 268), and enabled investigation of the BOR activity of single-entities, as well as their cluster size effects. Single NPs displayed a large variation in peak currents for direct BOR activity (from 0.12 nA to 0.62 nA at 0.05 V *vs.* SCE), which could not be explained by the size of the NP. The variation of several key electrochemical parameters could be determined from the single SECCM scan, as well as how their values and distributions changed as a function of cluster size. Abnormal electrochemical activities were also displayed by a proportion of single-NPs (17.4%, *N* = 178), which did not show clear peaks associated with the direct and indirect BOR reactions, as seen in the macroscopic CV. Many of the parameters taken from the BOR CV, such as *E*_onset_ and *Q*_forward_, were able to be statistically correlated with each other. Each parameter could be correlated with every other parameter, not just for one CV but for all potential cycles in a measurement, and for single NPs as well as each cluster size, allowing changes in parameter correlation to be visualised both between cycles, and as a function of cluster size increase. This is a clear demonstration of the ability of SECCM to generate statistically significant electrochemically-informative datasets from a single experiment. The most substantial finding of this work is that a significant proportion of locations containing single NPs showed no BOR activity (67.4%, *N* = 120), despite the standard drop-casting protocol employed. A key suggestion for any single-entity researcher using SECCM, or any similar technique, is not to rely on activity data alone when identifying locations of such entities, as any encapsulated entities which do not show activity for the desired process will be overlooked in the analysis. The application of this technique with well-defined structures, with a focus on full-dataset utilisation and exploration, is a clear future direction for single-entity research with SECCM.

## Data availability

Data will be made available on request.

## Author contributions

LFG and CLB provided experiment conceptualisation. LFG conducted the data curation, formal analysis, investigation, and writing. AMF guided methodology. CLB and AMF provided supervision.

## Conflicts of interest

The authors declare that they have no known competing financial interests or personal relationships that could have appeared to influence the work reported in this paper.

## Supplementary Material

SC-015-D4SC00676C-s001

SC-015-D4SC00676C-s002
