# Supplementary material for: Drop-cast gold nanoparticles are not always electrocatalytically active for the borohydride oxidation reaction
Source: Chem Sci. 2024 Apr 11;15(19):7243–58. doi: 10.1039/d4sc00676c (PMC11095372; doi:10.1039/d4sc00676c)
Supplement: SC-015-D4SC00676C-s001 [file SC-015-D4SC00676C-s001.pdf]

Electronic Supplementary Material (ESI) for Chemical Science.
This journal is © The Royal Society of Chemistry 2024

Supporting Information for:

Drop-cast Gold Nanoparticles are not Always Electrocatalytically Active for the Borohydride Oxidation Reaction

Lachlan F. Gaudin[a], Alison M. Funston[a,b], and Cameron L. Bentley[a,*]

[a]*School of Chemistry, Monash University, Clayton, 3800 VIC, Australia*

[b]*ARC Centre of Excellence in Exciton Science, Monash University, Clayton, 3800 VIC, Australia*

***Corresponding author**: cameron.bentley@monash.edu

Contents

Section S1. Characterisation of AuNP@HOPG electrode 3

Section S2. Supplementary Data for the SECCM Scan..4

Section S3. Supplementary Statistical Evaluations 7

Section S4. Movie captions 18

Section S1. Characterisation of AuNP@HOPG electrode

Table S1.1. NP counts, spatial densities, and nearest neighbour distances (NND) for a collection of ten sampled regions of the AuNP@HOPG surface. Images were collected via SEM, with an area of ~1150 μm^2.

Image Number	NP Count	Spatial Density (μm^{-2})	NND (μm)	NND standard deviation	NND %RSD
1	15	0.0130	4.95	116.0	23.4
2	18	0.0157	3.66	112.3	30.7
3	33	0.0287	2.31	81.5	35.2
4	19	0.0165	2.57	113.8	44.2
5	33	0.0287	1.80	91.0	50.6
6	52	0.0452	1.14	57.5	50.5
7	41	0.0357	1.74	74.7	42. 8
8	41	0.0357	1.29	53.5	41.3
9	41	0.0357	1.47	67.3	45.7
10	35	0.0304	1.60	75.1	46.8

Table S1.2. Totalled/Averaged results from Table S1.1

	Total NP count	Average Spatial Density	Average NND	Average NND standard deviation	Average NND %RSD
**Totals /Averages**	328	0.0285	2.26	84.3	41.12

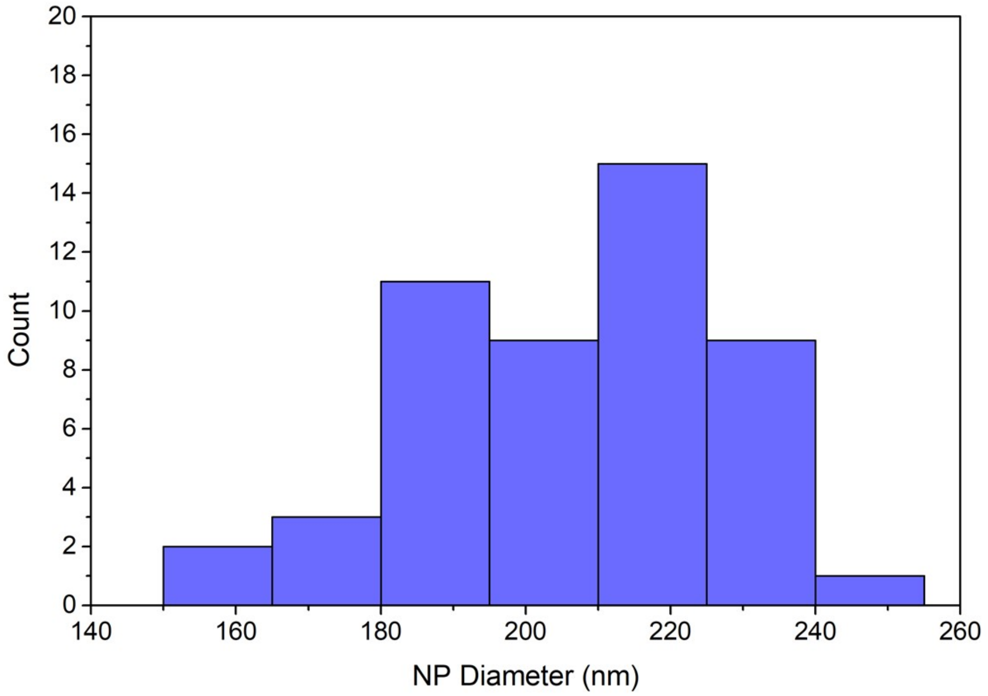

Figure S1.1. Distribution of NP diameters from a selection of 50 NPs on the AuNP@HOPG electrode.

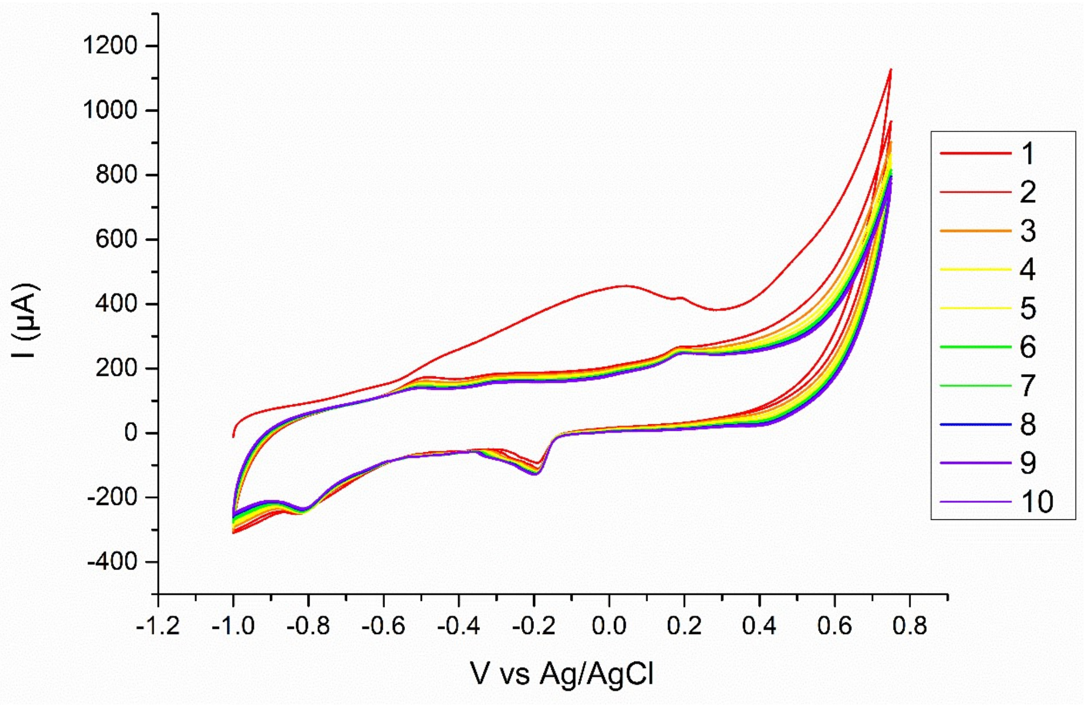

Figure S1.2. 10 CV cycles of electrochemical pre-treatment conducted on the AuNP@HOPG electrode in 0.1 M NaOH with a Pt counter electrode and a leakless Ag/AgCl reference electrode, performed at a scan rate of 0.5 Vs^{-1}.

Section S2. Supplementary Data for the SECCM Scan

Table S2.1. Total numbers and classifications of nanoparticle "type" from the SECCM scan featured in this work.

Number of NPs at location (total #)	Classification	Count (% in cluster size)
Single NPs (178)	Normal	27 (15.1)
	Abnormal	31 (17.4)
	Inactive	120 (67.4)
2 (49)	Normal	15 (30.6)
	Abnormal	12 (24.5)
	Inactive	22 (44.9)
3 (28)	Normal	9 (32.1)
	Abnormal	9 (32.1)
	Inactive	10 (35.7)
4 (10)	Normal	4 (40.0)
	Abnormal	3 (30.0)
	Inactive	3 (30.0)
5 (3)	Normal	1 (33.3)
	Abnormal	2 (66.6)
	Inactive	0 (0.00)

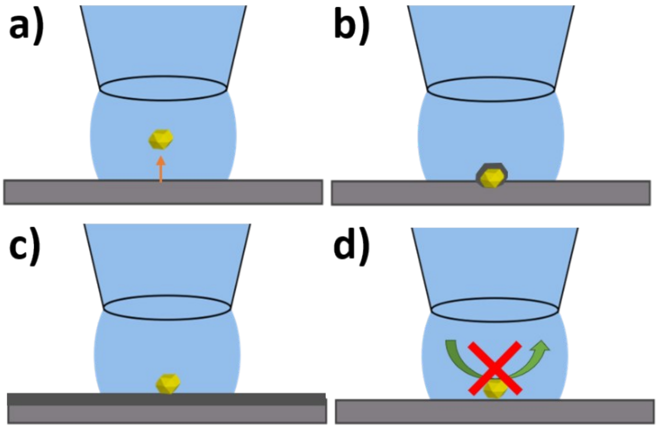

Figure S2.1. Diagrams of potential causes for particle abnormality and inactivity, **a)** particle detachment from surface, **b)** particle passivation by adsorbates, **c)** particle-substrate contact disruption, and **d)** intrinsically inactive particle.

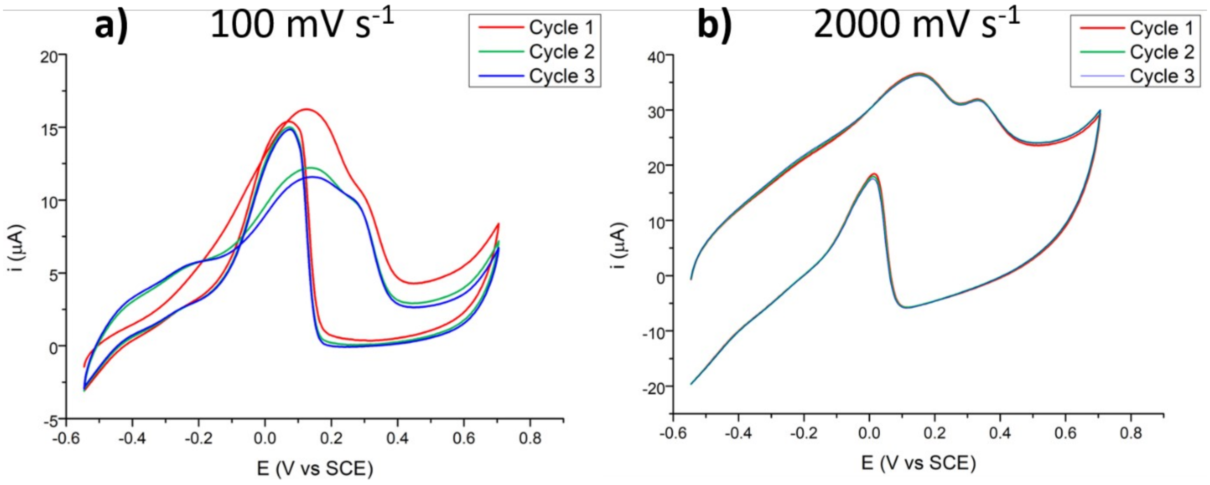

Figure S2.2 Comparison of sweep rate on macroscopic BOR activity. CVs were collected on a AuNP@GC macrodisk (3 mm diameter) following the exact drop casting procedure as for the HOPG sample. CVs were collected in 0.1 M NaOH containing 5 mM $NaBH_4$ purged with N_2 using a Pt wire counter electrode and a leakless Ag/AgCl reference electrode, later calibrated against an SCE reference. The 2000 mV/s CVs were collected one at a time, with electrolyte agitation (stirring) conducted between scans to simulate a pseudo-steady-state response, as generated in the SECCM experiment.

Section S3. Supplementary Statistical Evaluations

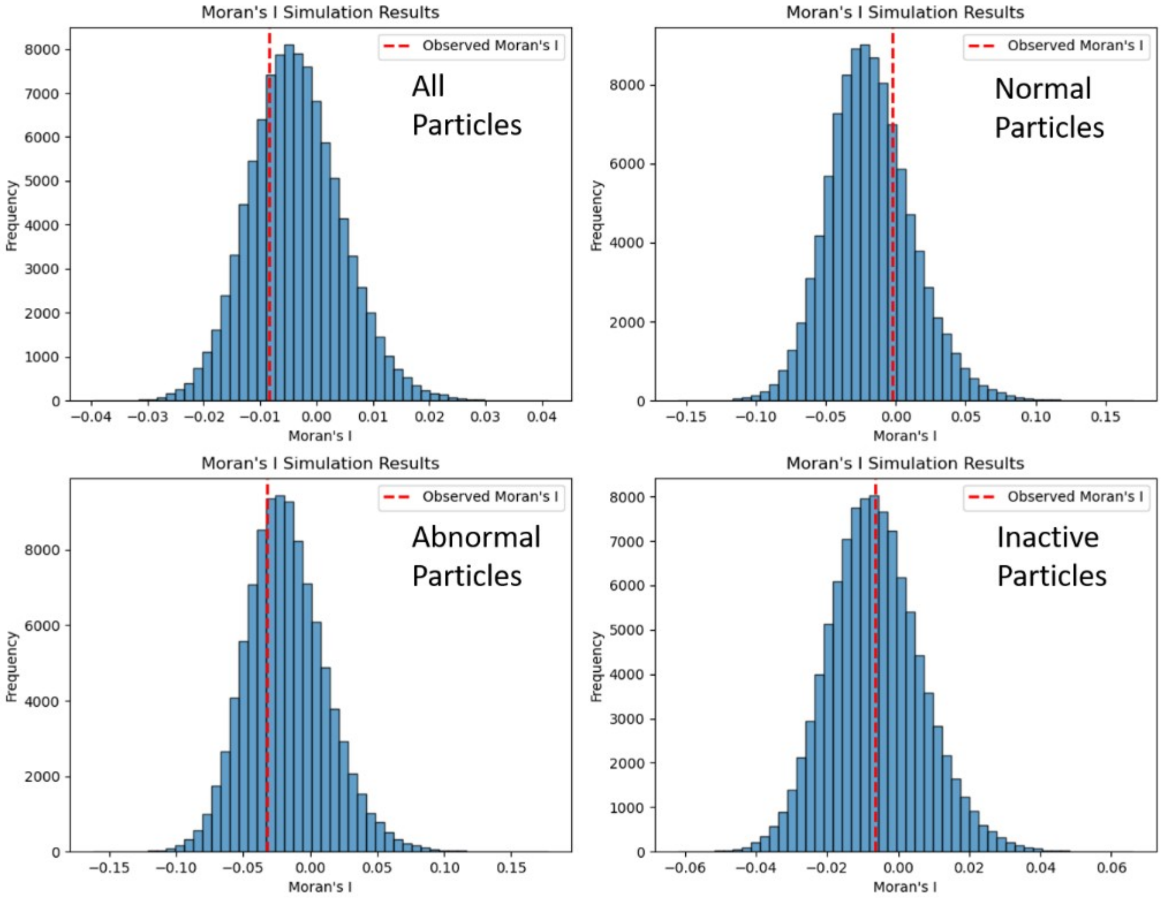

Figure S3.1. Calculated Moran's I and Monte Carlo Simulations for each of the four classifications of NP in the SECCM scan used in this work. Red line indicates the calculated value for the actual particle distributions. Monte Carlo simulations were performed with 100,000 randomly-generated permutations of the same size as the actual dataset.

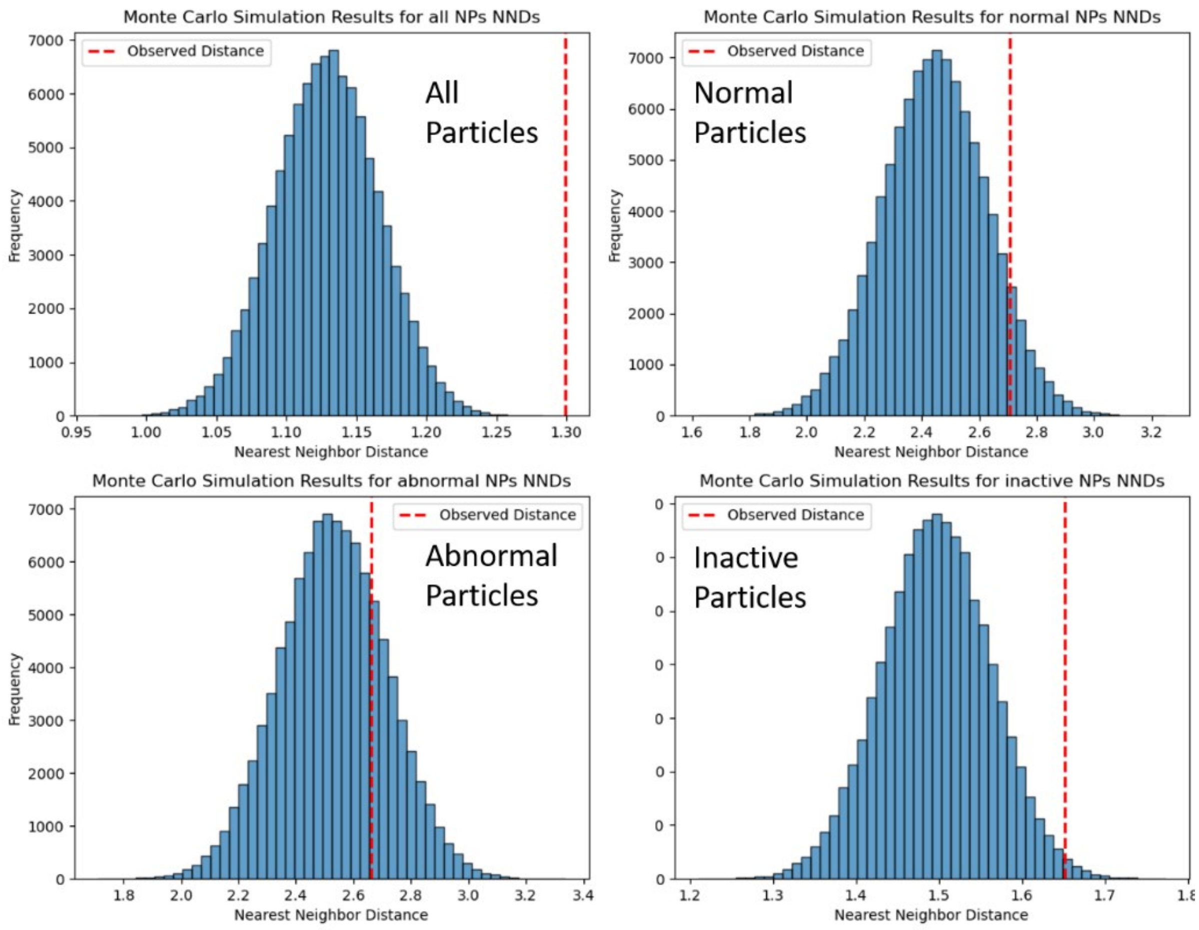

Figure S3.2. Calculated Nearest Neighbour Distances (NND) and Monte Carlo Simulations for each of the four classifications of NP in the SECCM scan used in this work. Red line indicates the calculated value for the actual particle distributions. Monte Carlo simulations were performed with 100,000 randomly-generated permutations of the same size as the actual dataset.

Table S3.1 In-depth definitions of the shorthand for the electrochemical CV parameters used in the text.

Shorthand	Definition
E_{onset}	Potential at which the current value reaches 4% of the maximum current
i_{onset}	Current value at the E_{onset}
E_{a1}	Potential of the first forward peak (a_1)
I_{a1}	Peak current of the first forward peak (a_1)
E_{a2}	Potential of the second forward peak (a_2)
I_{a2}	Peak current of the second forward peak (a_2)
$E_{off, forward}$	Potential at which the current is at a local minimum after the a_2 peak
E_{c1}	Potential of the first reverse peak (c_1)

I_{c1}	Peak current of the first reverse peak (c_1)
$E_{off, reverse}$	Potential at which the current returns to the i_{onset} value
$Q_{forward}$	Area under the CV on the forward sweep
$Q_{reverse}$	Area under the CV on the reverse sweep

Input Data

	Data	Range
Normal	[Book1]Sheet1!A"Normal"	[1*:14*]
Abnormal	[Book1]Sheet1!B"Abnormal"	[1*:12*]
Inactive	[Book1]Sheet1!C"Inactive"	[1*:21*]

Descriptive Statistics

	N Analysis	N Missing	Mean	Standard Deviation	SE of Mean
Normal	14	0	0.33857	0.70531	0.1885
Abnormal	12	0	0.2325	0.35871	0.10355
Inactive	21	0	0.1419	0.41495	0.09055

One Way ANOVA

Overall ANOVA

	DF	Sum of Squares	Mean Square	F Value	Prob>F
Model	2	0.32616	0.16308	0.63355	0.53548
Error	44	11.32612	0.25741		
Total	46	11.65229			

Null Hypothesis: The means of all levels are equal.
Alternative Hypothesis: The means of one or more levels are different.
At the 0.05 level, the population means are not significantly different.

Fit Statistics

R-Square	Coeff Var	Root MSE	Data Mean
0.02799	2.26887	0.50736	0.22362

Means Comparisons

Tukey Test

	MeanDiff	SEM	q Value	Prob	Alpha	Sig	LCL	UCL
Abnormal Normal	-0.10607	0.19959	0.75157	0.85636	0.05	0	-0.59017	0.37803
Inactive Normal	-0.19667	0.17506	1.58881	0.50495	0.05	0	-0.62125	0.22792
Inactive Abnormal	-0.0906	0.1836	0.69783	0.8748	0.05	0	-0.53591	0.35471

Sig equals 1 indicates that the difference of the means is significant at the 0.05 level.
Sig equals 0 indicates that the difference of the means is not significant at the 0.05 level.

Homogeneity of Variance Test

Levene's Test(Absolute Deviations)

	DF	Sum of Squares	Mean Square	F Value	Prob>F
Model	2	0.64947	0.32473	2.75984	0.07428
Error	44	5.1772	0.11766		

At the 0.05 level, the population variances are not significantly different.

Figure S3.3. Results of the analysis of variance conducted on NP-containing locations with cluster size = 2.

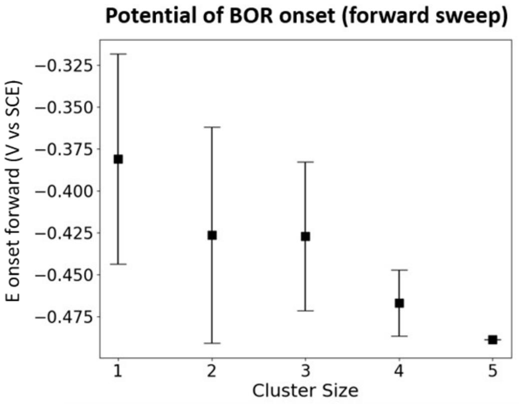
Potential of BOR onset (forward sweep)
E onset forward (V vs SCE)
−0.325
−0.350
−0.375
−0.400
−0.425
−0.450
−0.475
1 2 3 4 5
Cluster Size

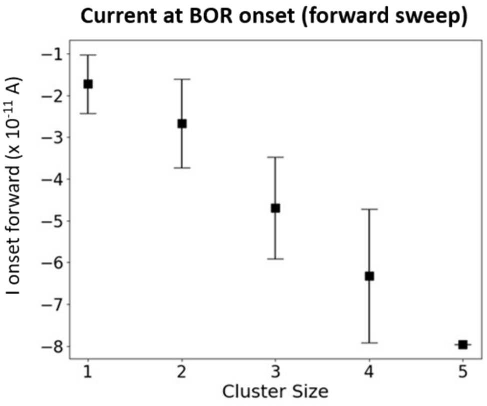
Current at BOR onset (forward sweep)
I onset forward (x 10^{-11} A)
−1
−2
−3
−4
−5
−6
−7
−8
1 2 3 4 5
Cluster Size

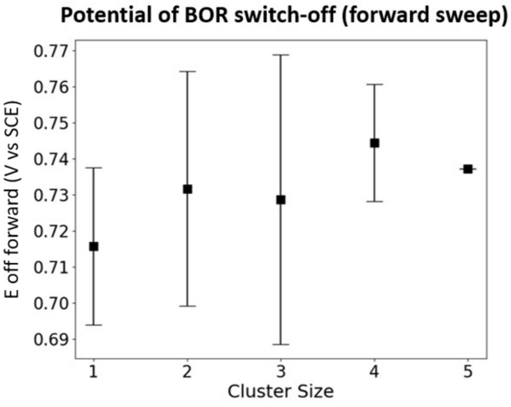
Potential of BOR switch-off (forward sweep)
E off forward (V vs SCE)
0.77
0.76
0.75
0.74
0.73
0.72
0.71
0.70
0.69
1 2 3 4 5
Cluster Size

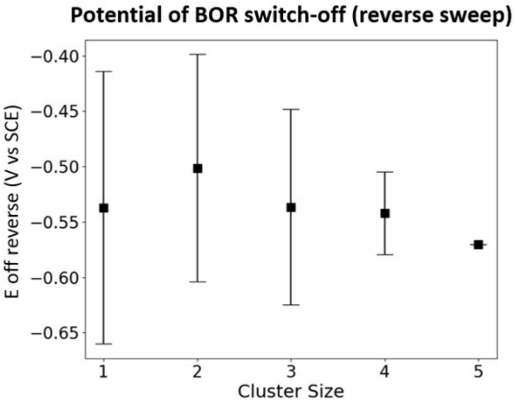
Potential of BOR switch-off (reverse sweep)
E off reverse (V vs SCE)
−0.40
−0.45
−0.50
−0.55
−0.60
−0.65
1 2 3 4 5
Cluster Size

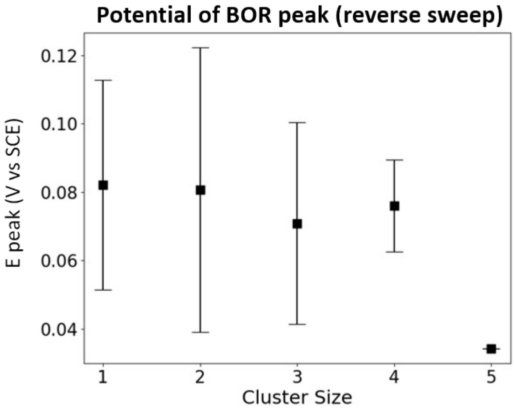
Potential of BOR peak (reverse sweep)
E peak (V vs SCE)
0.12
0.10
0.08
0.06
0.04
1 2 3 4 5
Cluster Size

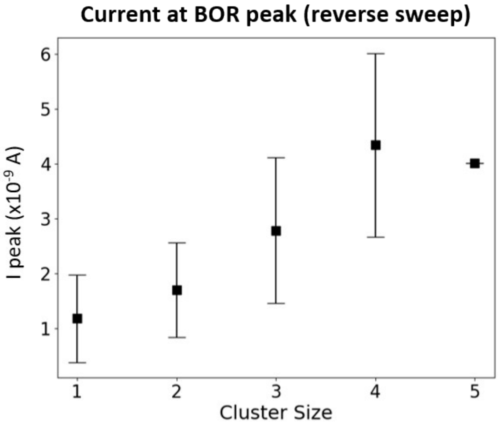
Current at BOR peak (reverse sweep)
I peak (x10^{-9} A)
6
5
4
3
2
1
1 2 3 4 5
Cluster Size

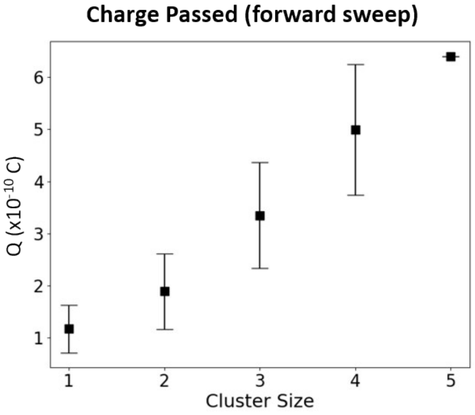
Charge Passed (forward sweep)
Q (x10^{-10} C)
6
5
4
3
2
1
1 2 3 4 5
Cluster Size

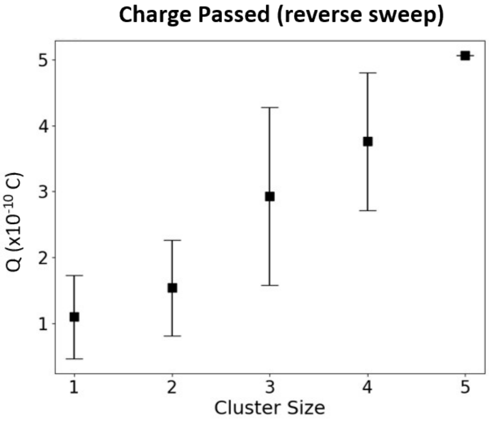
Charge Passed (reverse sweep)
Q (x10^{-10} C)
5
4
3
2
1
1 2 3 4 5
Cluster Size

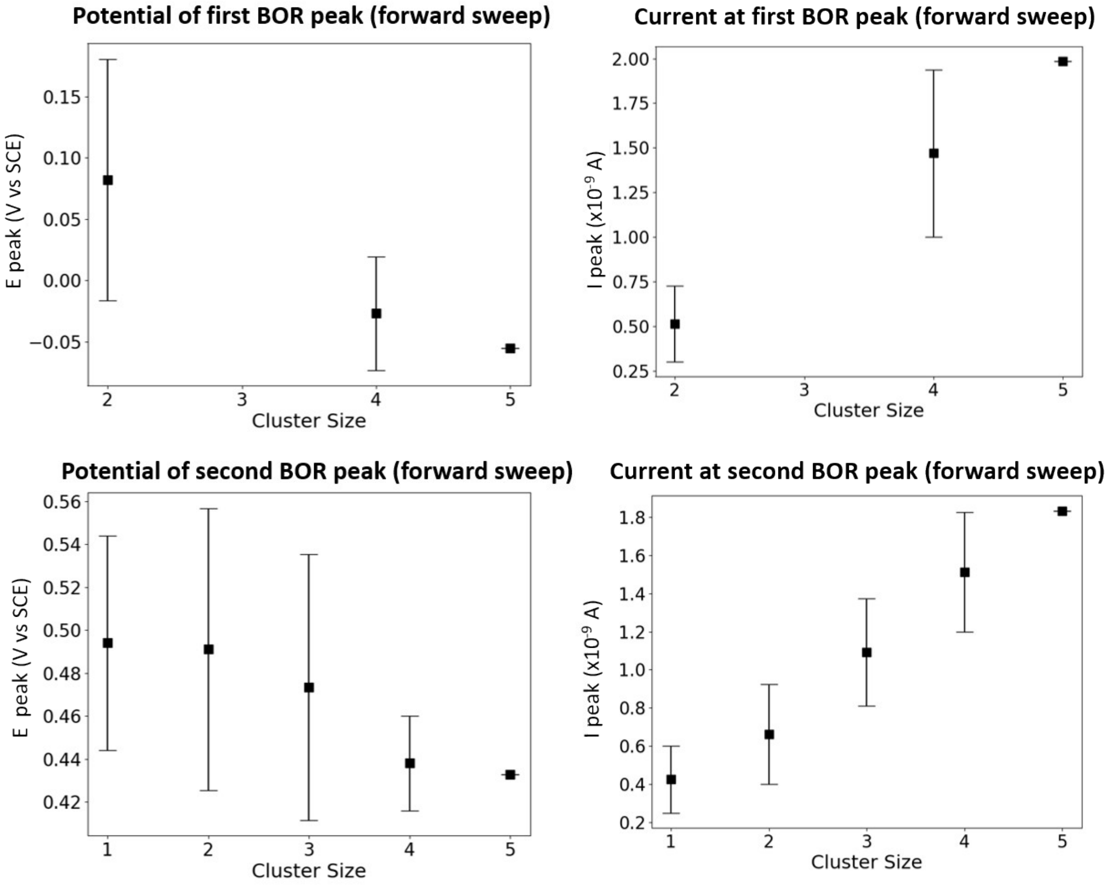

Figure S3.4. Indicative characteristics of the BOR CV as a function of particle cluster size, for the third CV cycle

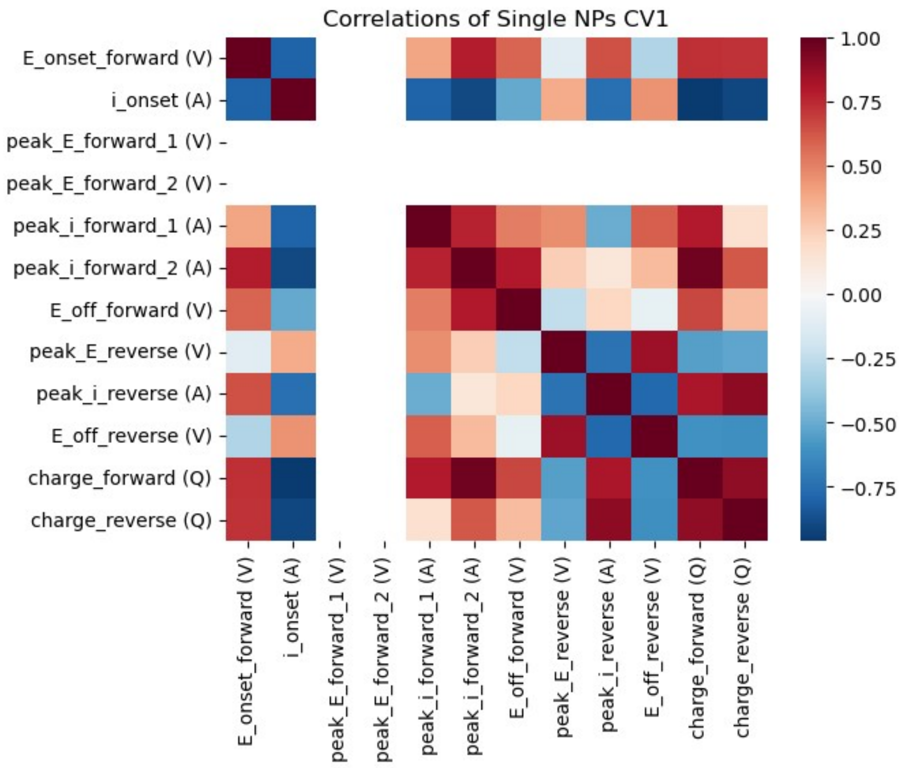

Figure S3.5. Pearson Coefficient heatmap for the 1st CV cycle of single particle locations. Empty columns and rows here represent lack of values due to the lack of the first BOR peak in the first cycle.

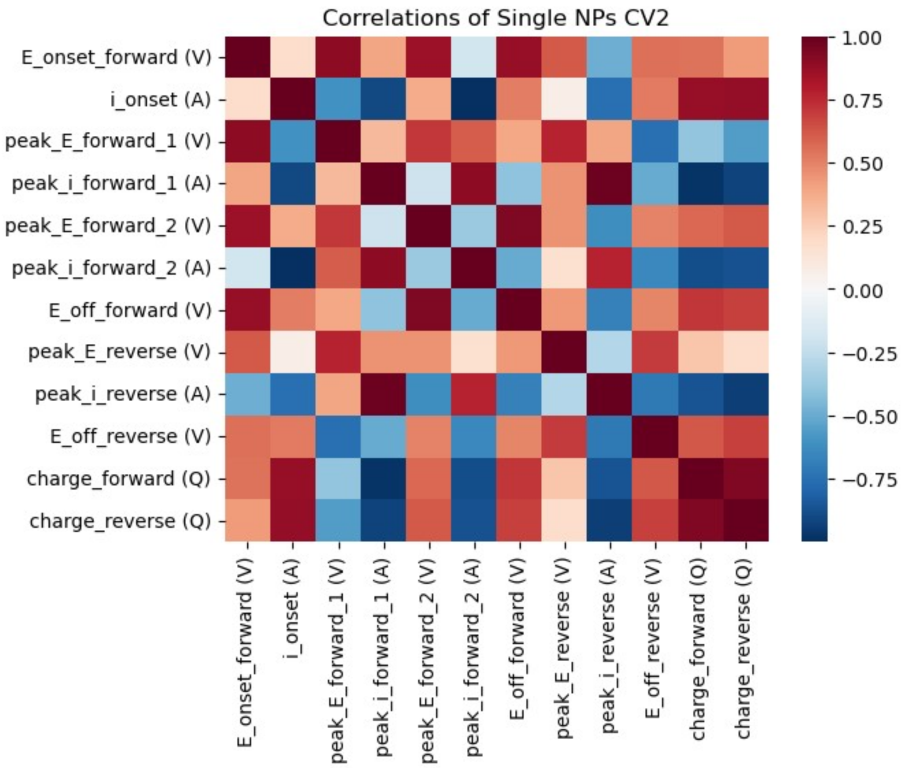

Figure S3.6. Pearson Coefficient heatmap for the 2nd CV cycle of single particle locations

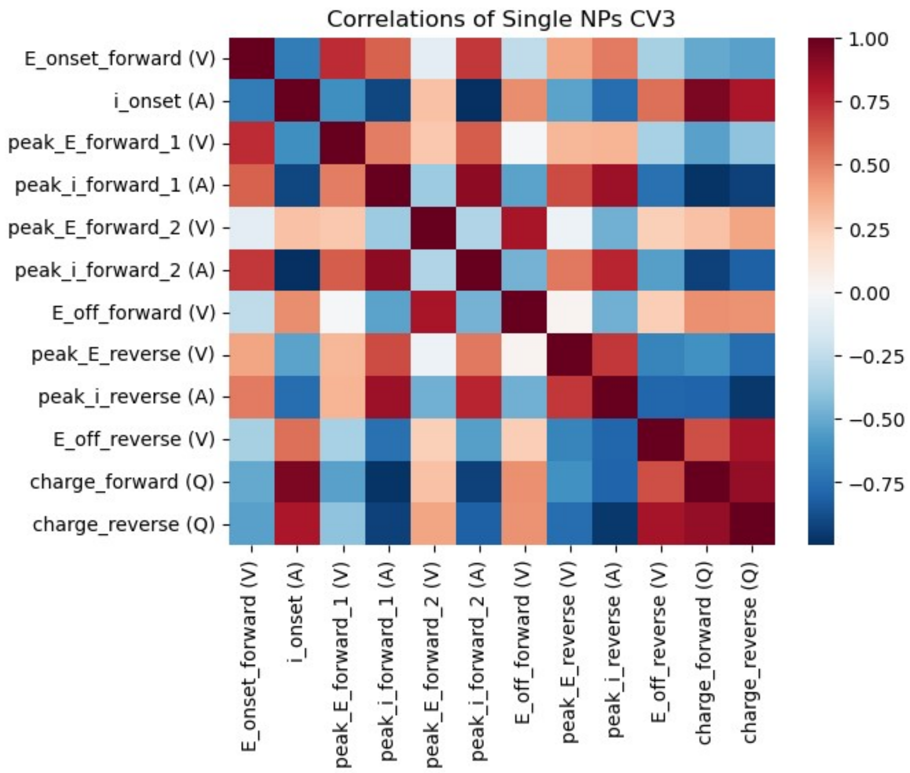

Figure S3.7. Pearson Coefficient heatmap for the 3rd CV cycle of single particle locations

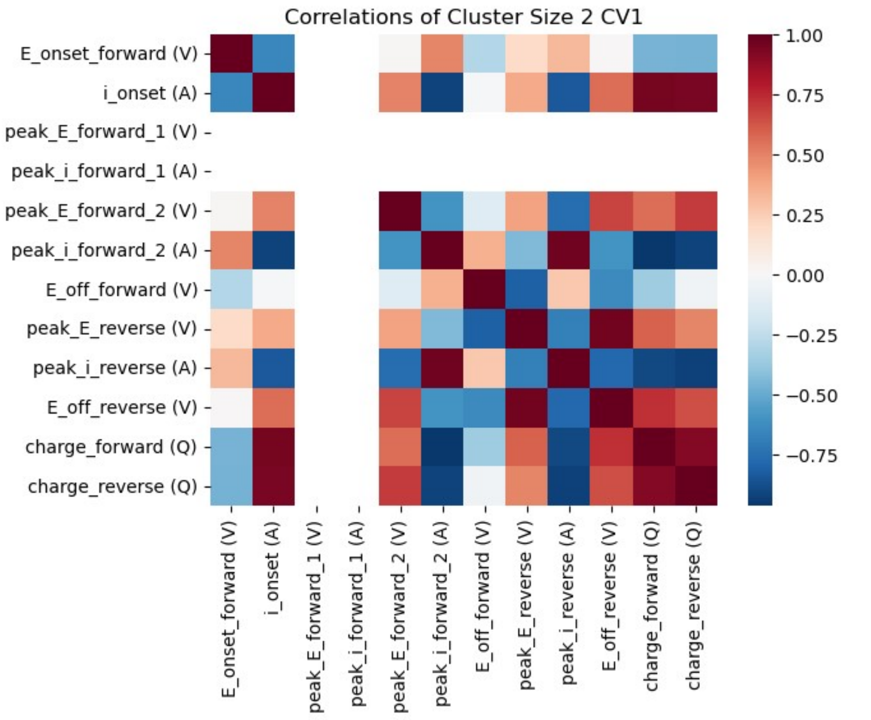

Figure S3.8. Pearson Coefficient heatmap for the 1st CV cycle of particle locations of cluster size 2. Empty columns and rows here represent lack of values due to the lack of the first BOR peak in the first cycle.

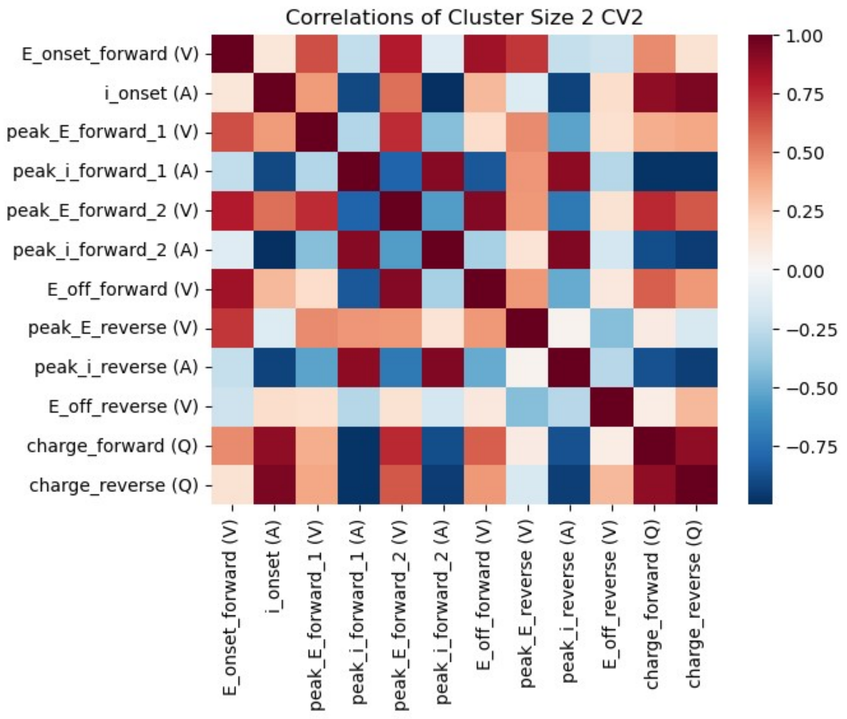

Figure S3.9. Pearson Coefficient heatmap for the 2nd CV cycle of single particle locations of cluster size 2.

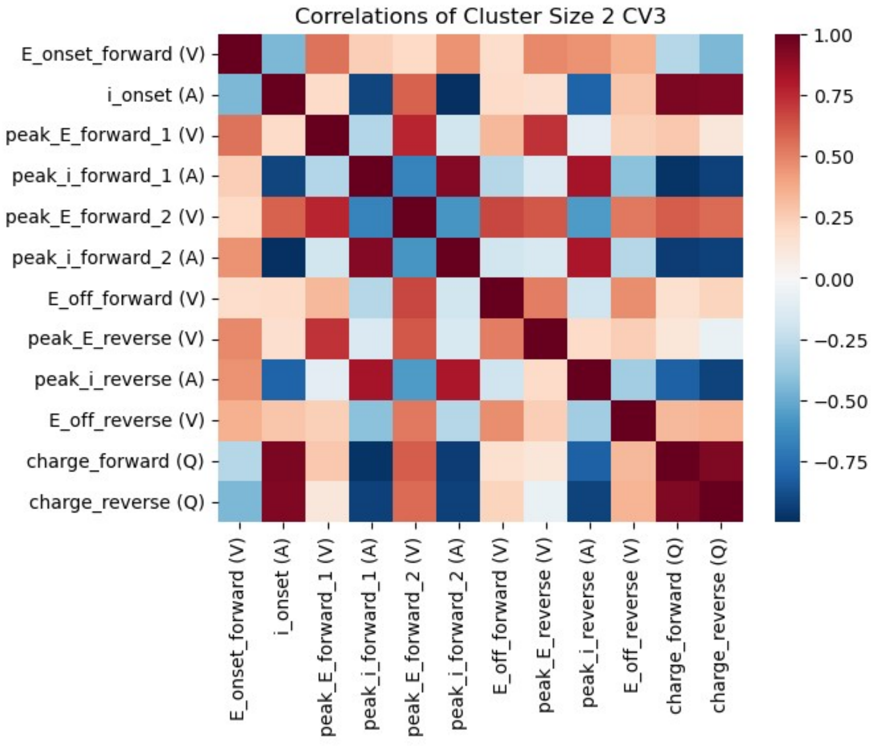

Figure S3.10. Pearson Coefficient heatmap for the 3rd CV cycle of single particle locations of cluster size 2

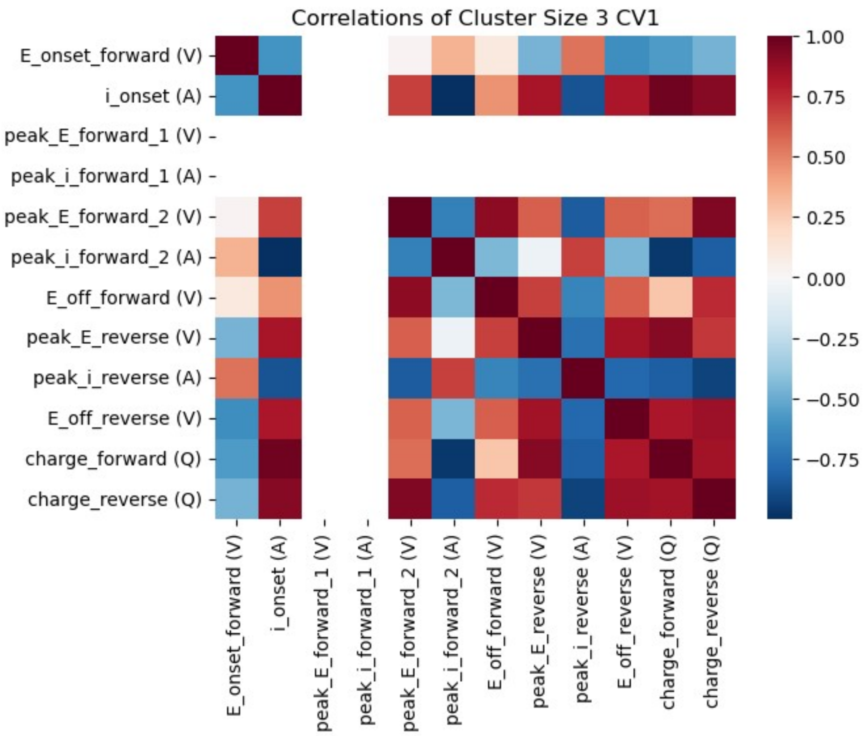

Figure S3.12. Pearson Coefficient heatmap for the 1st CV cycle of particle locations of cluster size 3. Empty columns and rows here represent lack of values due to the lack of the first BOR peak in the first cycle.

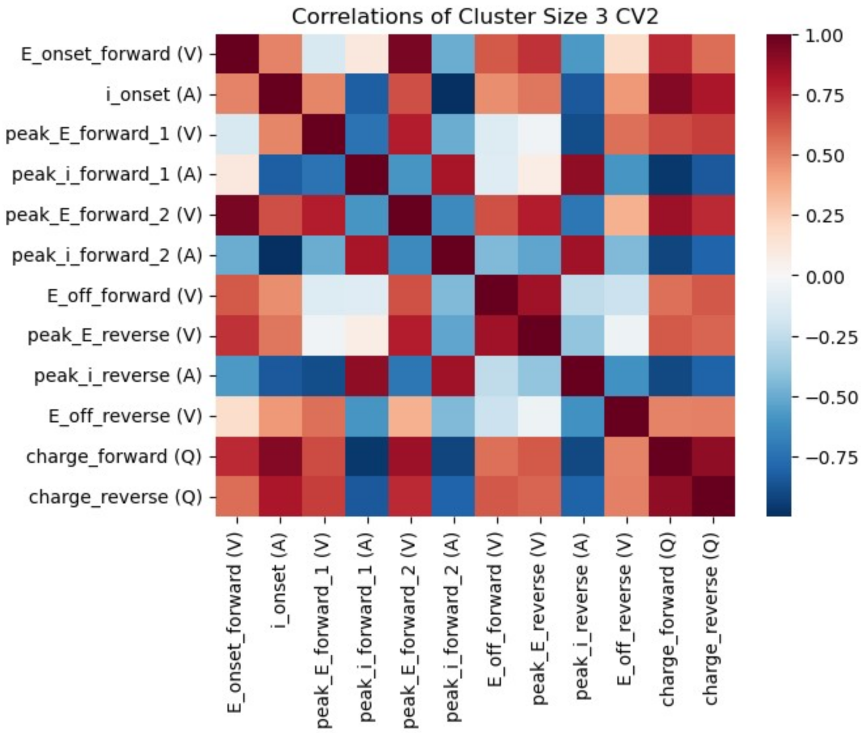

Figure S3.13. Pearson Coefficient heatmap for the 2nd CV cycle of single particle locations of cluster size 3.

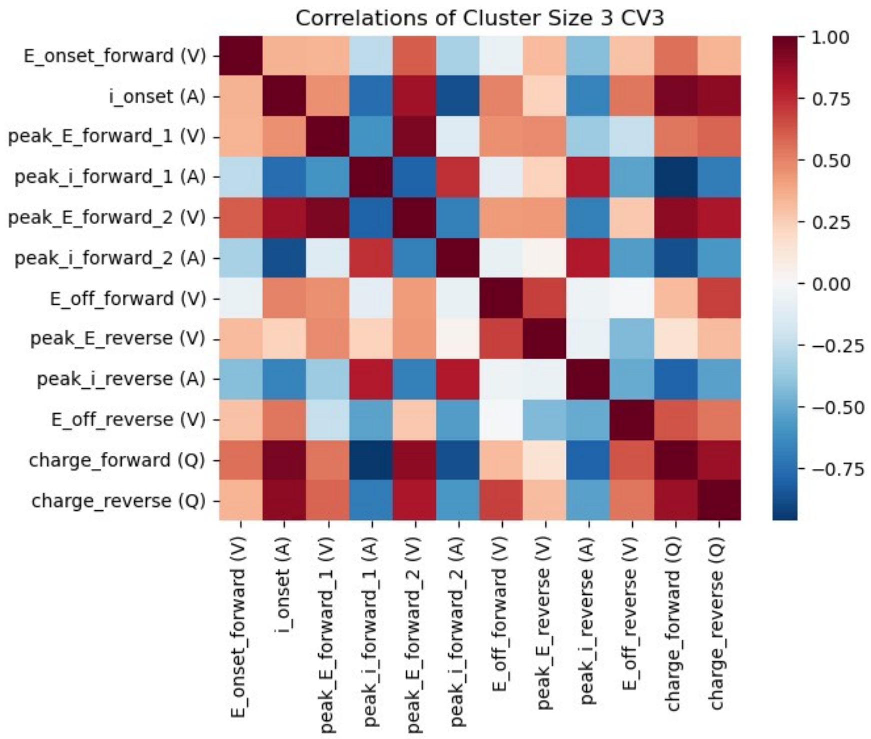

Figure S3.14. Pearson Coefficient heatmap for the 3rd CV cycle of single particle locations of cluster size 3

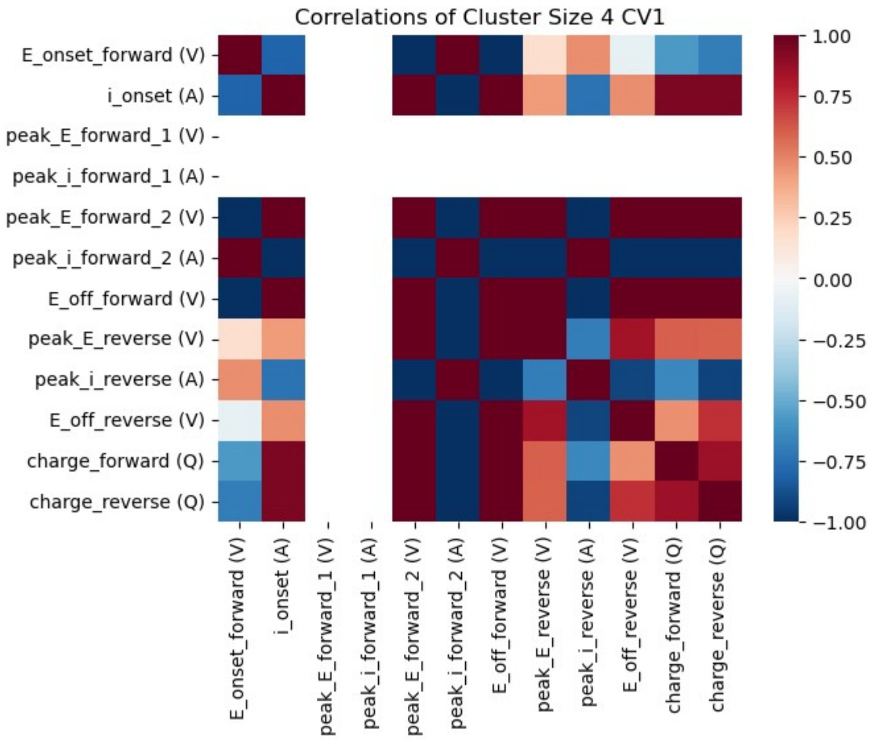

Figure S3.15. Pearson Coefficient heatmap for the 1st CV cycle of particle locations of cluster size 4. Empty columns and rows here represent lack of values due to the lack of the first BOR peak in the first cycle.

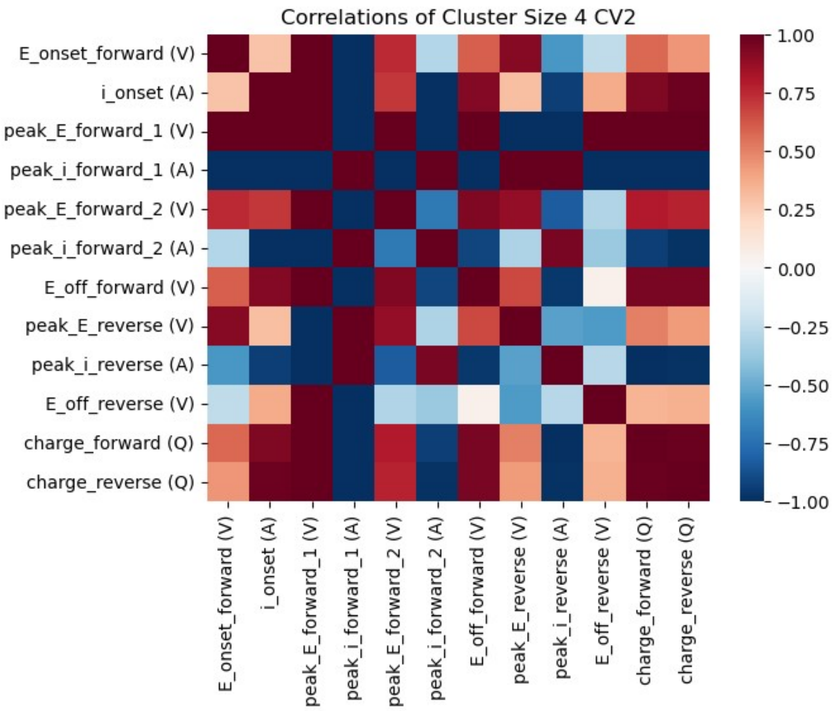

Figure S3.16. Pearson Coefficient heatmap for the 2nd CV cycle of single particle locations of cluster size 4.

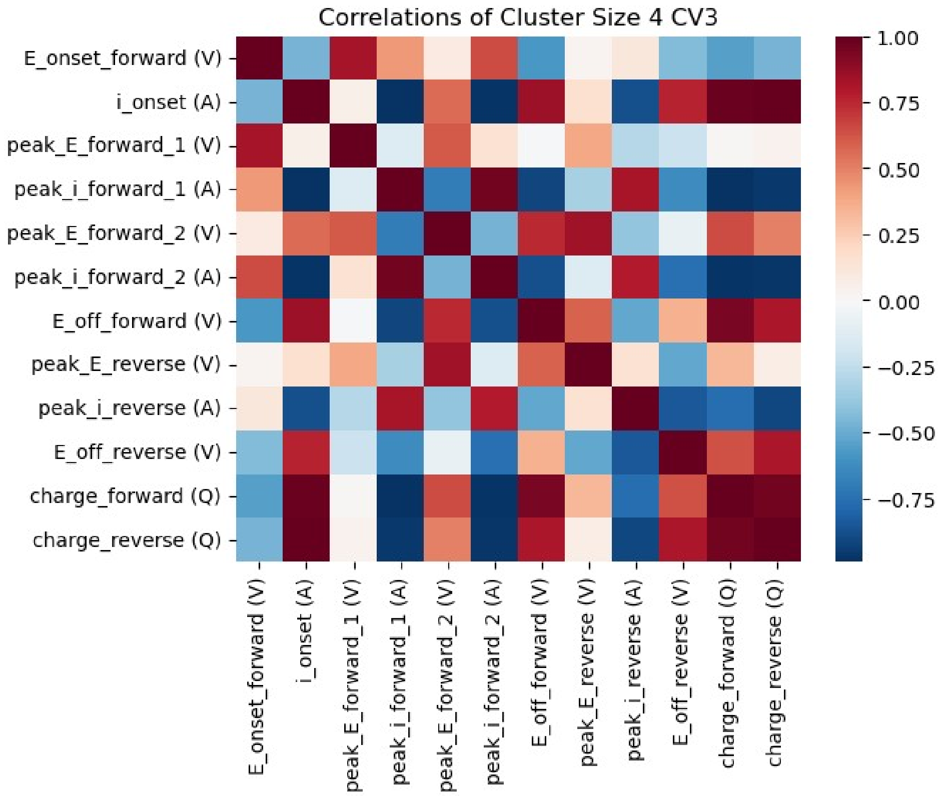

Figure S3.17. Pearson Coefficient heatmap for the 3rd CV cycle of single particle locations of cluster size 4

Section S4. Movie captions

Movie S1. Spatially-resolved electrochemical movie (1444 pixels over a 190 × 190 μm^2 scan area) obtained with the voltammetric hopping mode SECCM protocol, visualizing electrochemical activity at scan area 1 on the AuNP@HOPG sample. Experimental parameters were as follows: cyclic voltammogram (CV) waveform with a voltammetric scan rate (υ) = 2 V s^{-1}, approach voltage (E_a) = 0 V vs Ag/AgCl QRCE, initial potential (E_i) = –0.75 V vs Ag/AgCl QRCE and final potential (E_f) = 1.00 V vs Ag/AgCl vs QRCE (correction to SCE = +72 mV). The data shown is from the third CV cycle. The data presented are not interpolated. The data contained in this movie were used to construct all figures in the text.
